# Mechanism of Hyaluronic Acid Hydrolysis Catalyzed by Snake Venom Hyaluronidase

**DOI:** 10.1021/acs.jcim.5c02594

**Published:** 2026-01-27

**Authors:** Juliana Castro-Amorim, Maria J. Ramos, Pedro A. Fernandes

**Affiliations:** LAQV, REQUIMTE, Departamento de Química e Bioquímica, Faculdade de Ciências, 131674 Universidade do Porto, Rua do Campo Alegre, s/n, 4169-007 Porto, Portugal

## Abstract

Hyaluronidases are widely distributed in nature being ubiquitous in snake species (svHyal). They catalyze the hydrolysis of β-1,4-glycosidic bonds in hyaluronic acid, a critical constituent of the extracellular matrix. This facilitates the spread of venom toxins into the bloodstream, exacerbating tissue damage and systemic toxicity—a rationale for their common designation as “spreading factors”. While svHyals are not directly toxic, they substantially contribute to the morbidity and mortality associated with snakebite envenomation, the world’s most lethal neglected tropical disease. Despite their important role in tissue penetration, the atomic–level reaction mechanism of these enzymes remains poorly understood. To bridge this knowledge gap, we studied the chemical mechanism of the Hyal-1 enzyme isolated from the Puff Adder viper (*Bitis arietans*), likely the major contributor to snakebite mortality in sub-Saharan Africa. We evaluated two alternative mechanistic scenarios, based on different protonation states for the active site “assisting residue” (Asp110), and conducted umbrella sampling QM/MM MD simulations (PBE/DZVP-GTH-PBE: AMBER). Our findings indicate that the pathway starting from a neutral Asp110 yields an activation free energy barrier of 20.34 kcal·mol^–1^—nearly half that of the alternative pathway that considers an ionised Asp110. The deglycosylation step of the most favorable pathway yielded a free energy barrier of 13.94 kcal·mol^–1^. Our simulations also support an induced-fit mechanism for the svHyal/hyaluronic acid complex, with substrate distortion (chair → boat/skew-boat) favoring a conformation that closely mimics the transition state. This distortion, along with a prealignment of Glu112, lowers the activation free energy, enhancing the susceptibility of the glycosidic bond to nucleophilic attack. The results are likely transferable to all svHyal given their high degree of interspecific similarity (>90% sequence identity). This study highlights the importance of understanding mechanistics, including detailed stereoelectronic conformations and subsite-specific interactions, for the design of novel and effective inhibitors with broad clinical and biotechnological applications.

## Graphical abstract

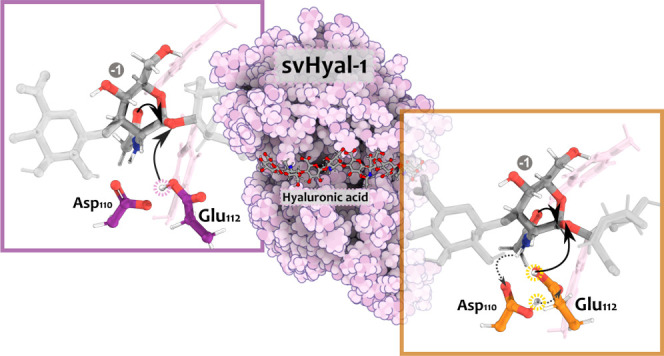

## Introduction

### The Health Burden of Snakebite Envenomation

Snakebite envenoming causes an estimated 81,000 to 138,000 deaths and more than 400,000 permanent disabilities (e.g., amputations) each year. Recognized by the World Health Organisation (WHO) as a “Category A Neglected Tropical Disease (NTD)”, snakebite envenoming represents a major public health and socio-economic issue, particularly in resource-limited tropical and subtropical regions like South and Southeast Asia, Latin America, and sub-Saharan Africa.^1,2^

In sub-Saharan Africa, the puff adder (*Bitis arietans*) is arguably the most medically significant snake due to its wide distribution, cryptic behavior, and potent venom.^3,4^

Following a snakebite, a large amount of venom (generally 100–350 mg in the case of the puff adder) may be injected into prey or human victims. This leads to severe local tissue damage at the injection site (i.e., hemorrhage, blistering, edema, and myo- and dermonecrosis) and widespread systemic toxicity (i.e., bleeding, coagulopathy, neurotoxicity, myotoxicity, and hypotension), which in severe cases can result in multiorgan failure and death.^5^ The outcome of envenomation depends on both the composition and amount of venom released, which are characteristic of the snake species, as well as the diffusion rate from the injection site to the bloodstream.^5,6^ This diffusion is largely facilitated by the breakdown of structural proteins, such as collagen, and glycosaminoglycans, such as hyaluronic acid, which are key components of the extracellular matrix.^5,7^

Snake venom is a complex cocktail containing dozens to hundreds of proteins and peptides, grouped into approximately ten principal protein families.^8^ The systemic dissemination of these toxins is primarily mediated by metalloproteases and hyaluronidases (svHyals), which act synergistically to disrupt the extracellular matrix integrity.^5,7^

As venom spreading is critical for the action of systemic toxins, this study focuses on the mechanism of the key spreading factor, the svHyal enzymes. We use the enzyme from the puff adder as a model system.

### Hyaluronic Acid: a Key Extracellular Matrix Scaffold

Hyaluronic acid (*M*_W_: 100–1000 kDa) is the simplest member of the glycosaminoglycan family, consisting of a tandem repeating unit of the [d-glucuronic acid-β1,3-*N*-acetyl-d-glucosamine-β1,4-]*n* disaccharide (i.e., GlcAβ(1 → 3)­GlcNAc units connected through a β(1 → 4) glycosidic linkage) (Figure S1).^5,9^ This linear, negatively charged polymer is widely found in the extracellular matrix of biological tissues, particularly in the skin, cartilage, connective tissue, and joints.^9,10^ Thus, as a key component of the extracellular matrix, hyaluronic acid plays vital roles in both physiological and pathological processes, including embryogenesis, angiogenesis, wound healing, tissue remodelling, tumor progression, and inflammation. Its remarkable versatility stems from its viscoelastic and biocompatible properties, supporting its use in musculoskeletal, ocular, dermatological, and regenerative medicine as a lubricant, structural matrix, and protective agent.^5,9,11^

### Snake Venom Hyaluronidase: a Neglected Spreading Enzyme

Hyaluronidases (Hyals) are enzymes that belong to the glycoside hydrolase family 56 (of 189 families) (EC 3.2.1.35), as classified by the Carbohydrate-Active Enzymes (CAZy) database^12^ (www.cazy.org, accessed 18 January 2025). While these enzymes are not inherently toxic, they facilitate systemic envenomation.^5,7,13^ Hyals catalyze the hydrolysis of hyaluronic acid, also known as hyaluronan, into smaller products, usually tetrasaccharides,^7,14^ thereby increasing tissue membrane permeability and facilitating the spread of high-molecular-weight toxic compounds into the bloodstream^15^ (Figure S2). This property has earned Hyals the title of “spreading factor”.^5–7,11^

These enzymes have been isolated from various sources, ranging from bacteria to mammals, and are nearly ubiquitous across snake venoms.^5,7,11^ The svHyal activity is generally higher in venoms from the Viperidae family, as elapid venoms are predominantly composed of low molecular weight toxins and hence do not require extensive degradation of the extracellular matrix^5,7^ (for an in-depth analysis of snake venom composition, please refer to refs 8 and 16 ). Nevertheless, the potential of inhibiting svHyals in snakebite therapeutics has been under-investigated for decades by the scientific community,^11^ overshadowed by the more abundant and highly toxic snake venom enzymes Zn-metalloproteinases, phospholipases A_2,_ and serine proteases, common to viperid venoms, and three-finger toxins, which are characteristic of elapid venoms.^5^

Although often overlooked in snakebite research, Hyals have long-standing clinical uses. Since the 1940s, they have been employed to enhance the diffusion of local anesthetics and are currently used in drug delivery, ophthalmology, orthopedics, and other fields.^6,9,15,17^ However, the role of Hyal in envenomation-related morbidity and mortality is increasingly recognized. Some experimental studies have reported inhibition of systemic effects by neutralization of Hyal’s activity,^18^ highlighting the potential of using high-affinity inhibitors targeting Hyal to treat snakebite. Hyal inhibition may also help regulate hyaluronic acid related pathologies, offering therapeutic potential as antiaging (maintaining skin’s hydration, elasticity, and structural integrity), anti-inflammatory (preventing formation of pro-inflammatory fragments), anticancer (suppressing tumor growth and angiogenesis), antibiotic (reducing bacterial tissue invasion) and contraceptive agents (preventing fertilization).

While the current animal-plasma-derived antivenom therapy can neutralize venom toxins, it is expensive and resource-demanding in rural areas and lacks effectiveness in preventing local tissue damage. This is because antibody-based therapeutics often do not reach the affected area in sufficient concentrations, especially if the venom toxins are already widespread. The early use of inhibitors that target svHyals may help preserve extracellular matrix integrity and delay systemic toxin spread.^7,9,15^ Therefore, these inhibitors could serve as first-aid agents against the consequences of ophidian incidents, ultimately improving the chances of survival of the envenomed victims.^7^ To devise strategies for developing such inhibitors, we must look into the structure and function of svHyals in detail.

### Structural Organization and Conserved Catalytic Architecture of svHyals

The UniProt database^13,19^ contains eight complete and curated amino acid sequences of viper venom Hyals from six different species (*B. arietans*, *Crotalus adamanteus*, *Echis ocellatus*, *Echis pyramidum leakeyi*, *Cerastes cerastes* and *Crotalus durissus terrificus*). However, none of their structures have yet been deposited in the PDB. In contrast, six X-ray structures of Hyals derived from wasp venom (PDB 2ATM.^20^) and bee venom (PDBs 1FCV, 1FCQ, 1FCU.,^21^ and 2J88)^22^ are deposited in the PDB, along with the human hyaluronidase (hHyal-1) crystal structure (PDB 2PE4.^23^). The cryo-EM structure of human sperm hyaluronidase (PH-20) (PDB 9JUB.^24^) with lower resolution data is also available. While the hHyal-1 is involved in tissue remodelling and pathological processes like tumor progression, the PH-20 plays a primary role in reproduction by facilitating sperm penetration through a hyaluronic acid layer surrounding the oocyte.^23,24^

Despite the absence of crystallographic information on svHyals, possibly due to their instability, rapid degradation, and relatively low concentration in venom samples, some have been successfully characterized experimentally through biochemical and structural studies.^9,13,25–27^

Sequence-homology analyses of the primary sequences indicate that svHyals share more than 93% sequence identity among them. The sequence identity with bvHyals is typically around 30%, and it increases to approximately 40% when compared to the human hyaluronidases hHyal-1 and PH-20 ^19^ (Figure S3). Additionally, those studies provided valuable insights into the svHyal structure, allowing for the mapping of structural features between svHyal Alphafold-predicted structure (AF-A3QVN9-F1) and its human, bee, and wasp homologues. Figure S3 displays the structural conservation among Hyal enzymes, showing that the major differences are limited to the N- and C-terminal regions. Both svHyal and hHyal-1 are believed to be globular, double-domain proteins. They feature an N-terminal catalytic TIM-barrel domain and a C-terminal epidermal growth factor (EGF)-like domain comprising 83 residues (Thr337-Ile426). This EGF-like domain starts with an extension of the α7/α8 helix (svHyal/hHyal-1) and contains three of the five disulfide bonds (Cys342–Cys353, Cys347–Cys404, and Cys406–Cys415), resembling the characteristic pattern of EGF-like domains.^10,23^ In addition to the helical extension, the EGF-like domain includes another helix (αI), a reverse turn β-hairpin (βII–βIII), a long two-stranded antiparallel β-sheet (composed of βI and βIV), and a turn at the C-terminal end.^10,13^ The EGF-like domain is thought to be important for regulatory processes.^13^ In contrast, bvHyals are single-domain enzymes that lack the EGF-like domain. Studies suggest that, like bvHyals, svHyals adopt a distorted (β/α)­7 triose phosphate isomerase (TIM) barrel fold.^10^ Unlike the classic TIM barrel formation, these enzymes contain only 7 β-strands rather than the typical 8, as the β2 strand found in hHyal-1 is missing in both svHyal and bvHyal.^7,10,28^

The active site of svHyals lies within a large, elongated groove, referred to as the hyaluronic acid-binding cleft. This cleft, primarily formed by positively charged and/or hydrophobic residues from the loops of the catalytic domain, is sufficiently large to accommodate at least an octasaccharide segment of the hyaluronic acid negatively charged groups and hydrophobic ring surfaces.^10,28^ The other two disulfide bonds are in the catalytic domain (Cys24–Cys317 and Cys188–Cys204) and are also present in bvHyal.^10^

Structural, site-directed mutagenesis, and kinetic studies have demonstrated the critical role of specific conserved active site residues for the enzymatic reaction—the catalytic Glu131/113/112 residue (hHyal-1/bvHyal-1/svHyal numbering) and the substrate-positioning residues Asp129/111/110, Tyr202/184/183, Tyr247/227/230 and Trp321/301/315.^10,14,28,29^ Their mutation either completely abolished enzymatic activity or reduced catalytic efficiency.^14,29^ These residues are strictly conserved across the different taxonomic groups, highlighting their structural and mechanistic importance.^10,28^ From this point forward, we will use the numbering scheme of the *B. arietans* Hyal-1 structure (svHyal) (Figure 1).

**Figure 1:**
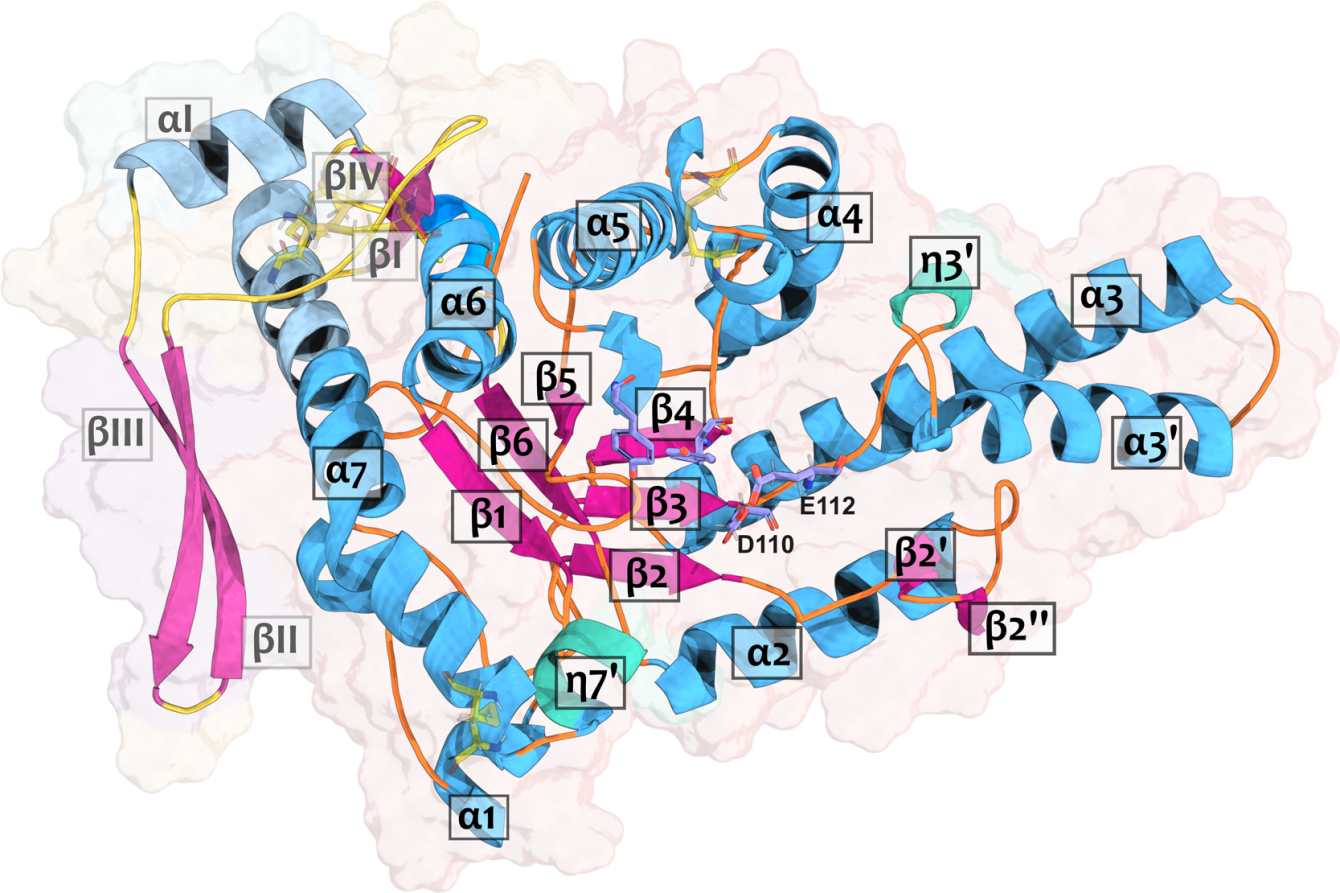
Cartoon representation of the svHyal tertiary structure obtained using AlphaFold (AF-A3QVN9-F1). α-Helices (blue), β-strands (pink), loops (orange), the catalytic Glu112, and the stabilizing/assisting Asp110 (violet sticks) are represented. The C-terminal epidermal growth factor (EGF)-like domain missing in the bvHyal structure (extension of the α7, βI, βII, βIII, βIV, and αI) is also shown in lighter colors. Yellow sticks represent the disulfide bonds (two in the main domain and three in the EGF-like domain).

### Mechanistic Insights into Hyaluronic Acid Degradation by Hyals

Despite extensive studies on GH family enzymes, the specific reaction mechanism by which Hyals degrade hyaluronic acid remains incompletely understood at the atomic level.^19^ Structural studies of both bvHyal and hHyals have led Marković-Housley and colleagues^21^ to adopt the nonclassical substrate-assisted mechanism originally proposed by Wang and Withers^30^ (Figure 2).

**Figure 2:**
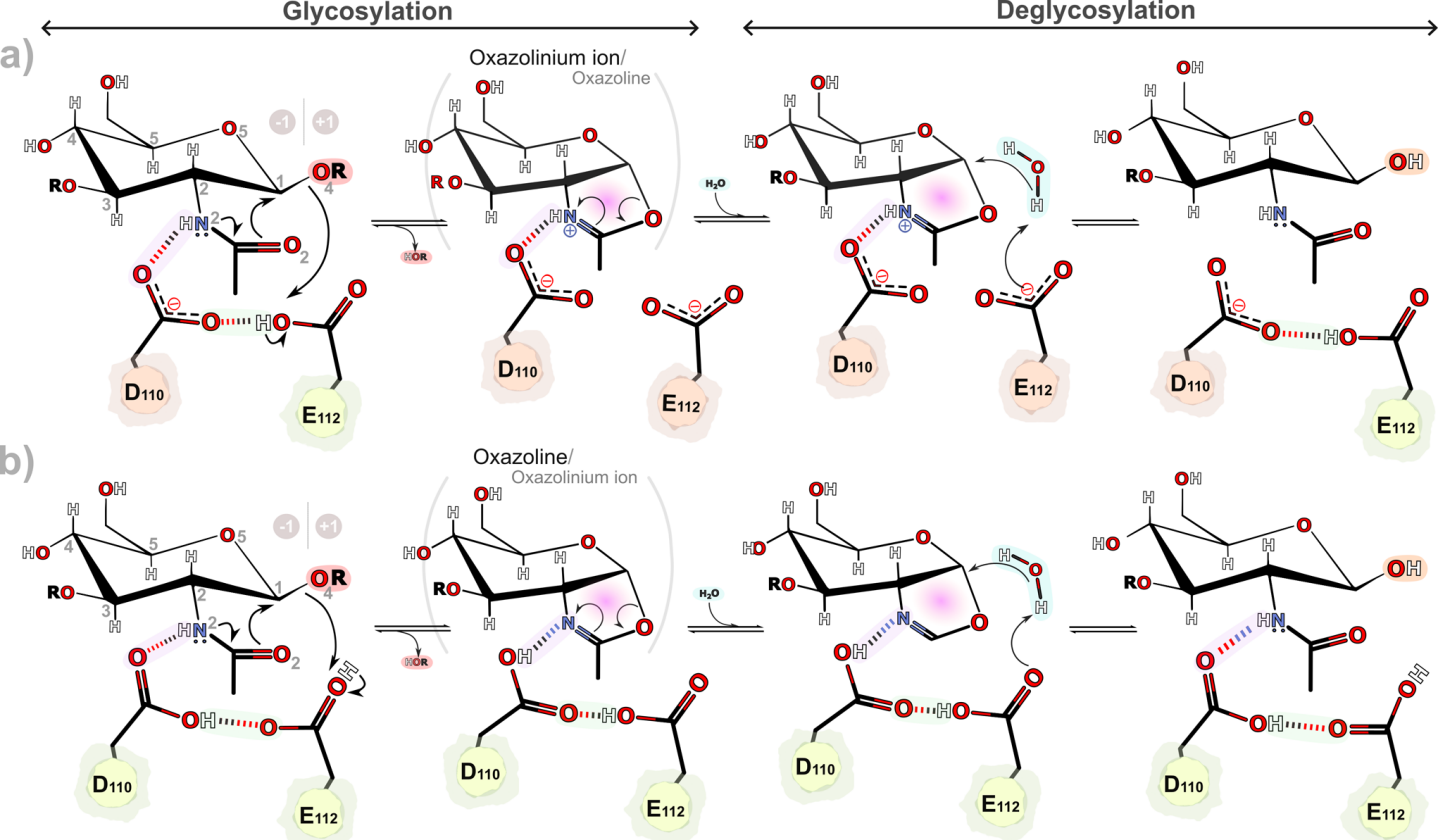
Schematic diagrams of the proposed mechanistic hypothesis for the svHyal. (a) Substrate-assisted mechanism, where the GlcNAc/acetamido carbonyl oxygen (O2) in the GlcNAc group of the hyaluronic acid substrate acts as a nucleophile to generate an enzyme-stabilized reaction intermediate. Glu112 serves as the catalytic acid/base residue, and the negatively charged Asp110 stabilizes the reaction intermediate (an oxazolinium ion or an oxazoline). (b) Another proposal of the substrate-assisted mechanism is where Glu112 protonates the glycosidic oxygen while being protonated by the neutral Asp110, which in turn receives a proton from the acetamido group of the hyaluronic acid substrate. The atomic numbering of the sugar ring is shown in gray in the first complex. The −1 and +1 subsites are highlighted with two light brown circles.

In this proposal, the reaction proceeds via an intramolecular nucleophilic attack in which the substrate’s GlcNAc carbonyl oxygen acts as nucleophile—distinguishing it from the classical one (e.g., lysozyme, GH22 and α-amylase, GH13), which is characterized by an enzyme nucleophile (usually a carboxylate residue) and the formation of a covalent glycosyl-enzyme intermediate (Figure S4).^31–33^ The substrate-assisted mechanism mainly involves the formation of a covalent oxazoline/oxazolinium ion intermediate (glycosylation step), followed by the hydrolysis of this intermediate (deglycosylation step) (Figure 2).^10,34^

The glycosylation step starts with a nucleophilic attack by the GlcNAc carbonyl oxygen (O2) on the anomeric carbon (C1) and the proton transfer from the catalyst Glu112 to the glycosidic oxygen atom (O4) at subsite −1/+1. This reaction leads to the cleavage of the β (1 → 4) bond, cyclization of the sugar ring, and the formation of a transient, short-lived oxocarbenium ion-like transition state. The nature of the resulting intermediate species at subsite −1 remains ambiguous, having been characterized as either an oxazolinium ion^31,35–37^ (GH18, GH20,^38^ GH123,^39,40^ GH25 ^40^) or an oxazoline (GH84).^39–41^ The main difference between these two possible intermediate species is the positioning of the proton, whether bound to the nitrogen atom of the GlcNAc unit (oxazolinium ion) or to the carboxylate oxygen atom of the assisting Asp110 residue (oxazoline).^42^ Finally, the cleaved sugar (from subsite +1) is released from the active site into the bulk solvent.

During deglycosylation, an incoming water molecule replaces the cleaved fragment. Here, Glu112 acts as a proton acceptor for this water molecule, preparing it for the next catalytic cycle.^10^ The water molecule, polarized by the Glu112 and Tyr183, then attacks the C1 atom of the reaction intermediate, breaking the covalent bond of the transient five-membered ring and completing the hydrolysis.^21,29^ Whether the intermediate reaction resembles an oxazoline or an oxazolinium ion ultimately affects the role of the Asp110 residue in this step. It can either cease to electrostatically stabilize the oxazolinium species or help return the abstracted proton to it.

This mechanism results in an overall retention of stereochemistry at the anomeric center via a double-displacement mechanism, first described by Koshland,^43^ indicating that the deglycosylation reaction is a reversal of the glycosylation.^10,34^

The released fragments serve as substrates for additional enzymatic cleavage, playing an important role in the remodelling dynamics of the extracellular matrix. Thus, Hyals are believed to degrade HA through a nonprocessive mechanism.^10,44^

In this study, we conduct umbrella sampling (US) QM/MM MD simulations to describe the reaction mechanism by which the African Puff Adder’s venom hyaluronidase-1 (svHyal) catalyzes the degradation of hyaluronic acid. QM/MM MD simulations have been widely used in mechanistic studies as they capture catalytic events that affect free energy profile and are often inaccessible to static QM/MM approaches. For example, Jitonnom et al.,^45^ demonstrated in their QM/MM MD study how transient interactions, substrate conformational motions, and protonation states shape the catalytic free energy during glycosidic bond cleavage of AXOS by the GH51 α-l-arabinofuranosidase from *T. xylanilyticus*. Similarly, Jitonnom et al.,^46^ applied QM/MM free energy simulations to a GH27 α-galactosidase, where conformational sampling provided mechanistic insights into transition-state stabilization. Thus, these and other studies exemplify how a QM/MM MD-based approach can provide a more complete mechanistic description of the most favorable catalytic pathway.

Here, we explore two different mechanistic scenarios based on the protonation states of the catalytic network residues: ionised Asp110 (Asp-COO^–^) or neutral Asp110 (Asp-COOH).

Moreover, we investigate the structural features of the reaction’s transition state and intermediate, and the conformational itinerary followed by the hyaluronic substrate pyranose ring occupying subsite −1 along the reaction pathway. The results clarify the chemical mechanism of svHyal and pave the way for the rational design of antienvenomation therapeutic inhibitors.

## Computational Methods

### Building the svHyal/Hyaluronic Acid Complex

In the absence of an experimental structure for the African puff adder Hyal-1 (SvHyal), we used its AlphaFold apoenzyme structure (Uniprot: A3QVN9, AlphaFold: AF-A3QVN9-F1-v4). The AlphaFold-derived model exhibited a very high average pLDDT score of 93.93, indicating a high confidence level in the predicted overall structure. The pLDDT further increased to 96.75 when excluding the signal peptide and the disordered C-terminal region. The protonation states of the titratable residues were assigned based on PROPKA predictions at pH 6.5 via the PDB 2PQR server^47^ (Table S1). The pH was chosen to reflect the slightly acidic conditions that mimic the localized microenvironment encountered during envenomation, where svHyal exhibits optimal activity for degrading hyaluronic acid. Based on p*K*_a_ predictions and visual inspection of the local chemical microenvironment, nearly all residues were predicted to retain their typical protonation states, except for the catalytic residues Asp110 and Glu112, whose p*K*_a_ values were high compared to standard values, at 8.81 and 6.08, respectively.

We generated two distinct scenarios to study the impact of specific protonation states of these catalytic residues on the catalytic mechanism. Either Asp110 was assigned as deprotonated (Asp-COO^–^ model) and Glu112 protonated, or both Asp110 and Glu112 were protonated (Asp-COOH model). Although this may appear to be a minor difference, it has significant implications for the reaction mechanism.^45,48^ The latter scenario was also tested by Coines et al.,^41^ Iino et al.,^42^ and Jitonnom et al.^36^ when studying enzymes from the Chitinase family.

Given the high structural similarity between bvHyal (PDB 1FCV.^21^) and svHyal-1 we transposed the bvHyal-bound tetramer product occupying the −4 to −1 subsites onto the svHyal-1 active site cleft by superimposing both structures. This also allowed us to map the active site’s conserved residues and identify potential interactions with the substrate. Afterward, we constructed a hyaluronic acid segment comprising a linear chain of four alternating *N*-acetylglucosamine (GlcNAc) and glucuronic acid (GlcA) disaccharide units using the Carbohydrate Builder module available on the GLYCAM web server (http://glycam.org/) and employed the GOLD docking software^49^ to bind it to the +1 to +4 subsites (Figures S5 and S6, Supporting Information—Section II, Methodology). Finally, we immersed the entire svHyal: hyaluronic acid complex in a cubic box of 105,534 TIP3P water molecules, ensuring a minimum distance of 10 Å between any enzyme atom and the box edges. The system’s charge was neutralized by replacing water molecules with chloride (Cl^–^) counterions. The final unit cell sizes were approximately 105.25 × 105.25 × 105.25 Å^3^ for both systems. The svHyal and the hyaluronic acid substrate were parametrized using the Amber ff14SB and GLYCAM_06j force fields, respectively. The water molecules and the Cl^–^ counterions were described using the TIP3P^50^ water model and the Joung–Cheatham parameters,^51^ respectively.

### Classical Molecular Dynamics (cMD) Simulations

We analyzed the svHyal-1: hyaluronic acid complex by running classical molecular dynamics simulations (cMD) using the GROMACS 2023 software package (https://manual.gromacs.org/). We employed the leapfrog^52^ MD algorithm with an integration time step of 2.0 fs. All intramolecular motions related to hydrogen bonding were constrained using the LINCS^53^ algorithm. To compute the long-range electrostatic interactions beyond the nonbonded interaction cutoff of 10.0 Å, we utilized the PME^54^ scheme with a PME order of 4 and a Fourier spacing of 0.16 Å, while truncating the Lennard-Jones interactions. The Verlet^55^ scheme was applied for neighbor searching. All simulations were performed under periodic boundary conditions in all directions. We maintained both protein and nonprotein coupling groups at a temperature of 310.15 K using the V-rescale^56^ thermostat with a coupling constant of 0.1 ps. During the *NPT* equilibration, the Parrinello–Rahman^57^ barostat was employed to ensure uniform scaling of the box vectors, with a reference pressure of 1.0 bar using an isotropic scheme and a coupling constant of 0.1 ps. Ultimately, we conducted MD simulations of 200 ns on 4 replicas for each model system. The root mean-square deviation (RMSD) of the protein backbone atoms stabilized at approximately 1.5 Å in the equilibrated structure when compared to the starting structure. A comprehensive description of the MD protocol can be found in Table S2 (Supporting Information—Section II, Methodology).

### Distance- and RMSD-Based Clustering Analysis

To identify productive conformations of the SvHyal-hyaluronic acid complex, we designed a custom TCL script to filter the MD trajectory. The first glycosylation step involves a proton transfer from Glu112 to the hyaluronic acid glycosidic bond. Therefore, appropriate interatomic distances and precise spatial orientations are required for the reaction to take place. As a result, potential reactive conformations (i.e., conformations where the active site residues are adequately preorganized to react in terms of orientation and distance) were identified using the following interatomic distance criteria: Oδ1 Asp110–H_2_N GlcNAc ≤ 3.0 Å, Oε2 Glu112–O4_glyc_ ≤ 4.0 Å, O2N GlcNAc–H Tyr230 ≤ 3.0 Å, and Oδ2 Asp110–Oε1 Glu112 ≤ 3.0 Å. These criteria ensured that the substrate was accurately positioned for catalytic interactions concerning svHyal active site residues. This is a necessary but not sufficient condition for the reaction to take place with a low free energy barrier, as the electrostatic field provided by the whole enzyme also contributes to the reaction. However, conformations that do not obey these necessary criteria are very likely nonreactive, as they lead to very high energy.^45,58,59^

These reactive conformations underwent further analysis to ensure structural diversity in the active site region. They were organized into distinct clusters based on the RMSD of residues’ backbone atoms within 4 Å of the catalytic residues and the hyaluronic acid substrate. The clustering was conducted using the GROMOS method, implemented in the GROMACS package. We chose the most prevalent and catalytically favorable conformation from the clustering analysis for further investigation. We stress that, despite starting the reactivity study from this conformation, the subsequent umbrella sampling ensures a relevant exploration of the phase space. Therefore, the choice of the initial conformation does not bias the results toward this conformation but instead avoids starting the subsequent reactivity study from a very unreactive starting point.

### QM/MM MD Simulations: Glycosylation Reaction Step

For the “Asp-COO^–^ model”, we prepared a QM layer that consisted of a total of 112 atoms, with a total charge of −3, a singlet spin multiplicity, and a unit cell of dimensions 24.10 Å × 22.35 Å × 22.66 Å. On the other hand, the “Asp-COOH model” contained 113 atoms, a total charge of −2, a singlet spin multiplicity, and a unit cell of dimensions 23.48 Å × 23.11 Å × 22.90 Å. Both models were composed of the Asp110 and Glu112 side chains, Tyr183 and Tyr230 phenol groups, and residues at subsites −2 (GlcA), −1 (GlcNAc), and +1 (GlcA). The MM layer, representing the remaining part of the system, was treated using the same force field applied in the cMD simulations.

We computed the interaction forces between the QM and MM layers using the QUICKSTEP^60^ and FIST modules of the CP2K software.^61^ For the QM layer, we applied DFT within the Gaussian plane wave formalism at the PBE/GPW level^62^ (with a plane-wave cutoff of 360 Ry),^63^ coupled with the DZVP basis set. Core electrons were described through GTH pseudopotentials.^64^

We performed an initial MM minimization of the system employing the LBFGS algorithm, followed by sequential QM/MM minimizations using both mechanical and electrostatic embedding schemes. We then equilibrated the system for 5 ps using the *NVT* ensemble with an integration time step of 1.0 fs. The temperature of 310.15 K was controlled through the CSVR thermostat.^56^ We used hydrogen atoms as “link” atoms to complete the valences at the boundary according to the IMOMM scheme.^65^ Long-range Coulomb interactions were approximated using the GEEP method,^66^ while the SPME^67^ method was employed to calculate electrostatic interactions. We set the scaling factors for 1–4 electrostatic and Lennard-Jones interactions to 0.8333 and 0.5, respectively. We did not apply restraints during the QM/MM MD simulations.

We used the equilibrated structure after equilibration to perform a 5 ps QM/MM steered molecular dynamics (sMD) simulation with an integration time step of 1.0 fs. We applied a harmonic force constant of 250.0 kcal mol^–1^ Å^–2^ along the reaction coordinate. We defined the reaction coordinate of the glycosylation step (RC_1_) as a single collective variable (CV_1_), represented by an additive distance function, where CV_1_ = *d*_1_ + *d*_2_. In both models (Asp-COO^–^ and Asp-COOH), CV_1_ represents the nucleophilic attack by the GlcNAc carbonyl oxygen (O2) on the C1 carbon (*d*_1_ = O2 GlcNAc – C1 GlcNAc), and protonation of the glycosidic oxygen atom (O_glyc_) by the catalytic glutamic acid (Glu112) (*d*_2_ = H Glu112 – O4_glyc_) (Figure 3). The initial sum of these distances, approximately 7.0 Å, was narrowed to the desired final sum of 2.3 Å, representing the combined decrease implied during proton transfer and nucleophilic attack until the new chemical bonds were formed with typical covalent bond lengths. We also calculated the atomic charges for the REACT, TS1, and INT1 structures of the Asp-COOH glycosylation step using the Hirshfeld^68^ scheme.

**Figure 3:**
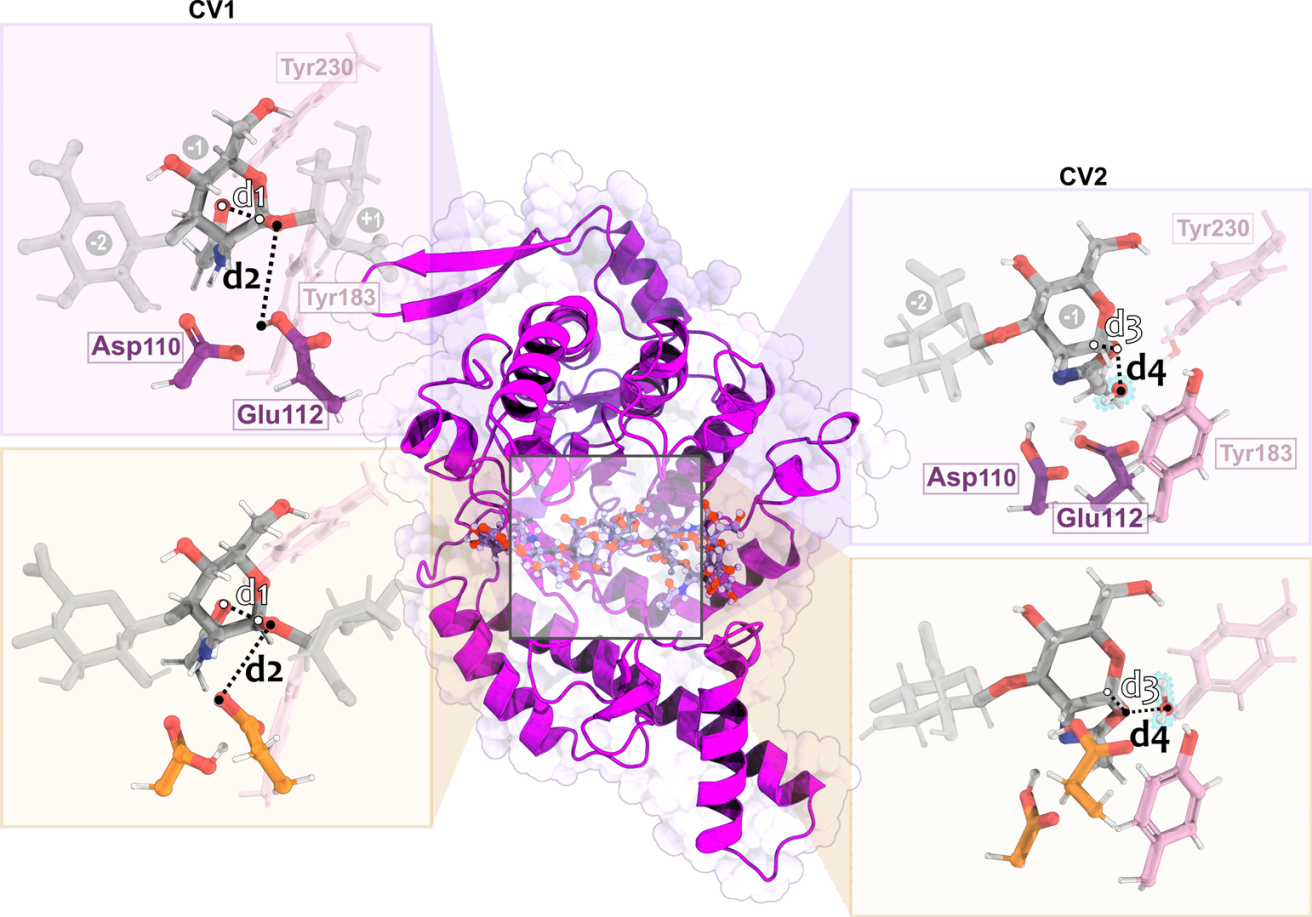
Cartoon representation of the svHyal structure, highlighting the hyaluronic acid binding site. (Left) A close-up view of the QM layer and the collective variable used to model hydrolysis of the β-1,4-glycosidic bond: reaction coordinate (RC_1_; CV_1_) used to describe the glycosylation step (dashed lines), CV_1_ = *d*_1_ + *d*_2_; (Right) A close-up view of the QM layer and the reaction coordinate (RC_2_; CV_2_) used to describe the deglycosylation step (dashed lines), CV_2_ = *d*_3_ – *d*_4_. The Asp–COO– (purple) and Asp-COOH (orange) models are represented by purple and orange sticks, respectively. The water molecule in CV_2_ is highlighted in blue.

### Preparation of the Reaction Intermediate

We retrieved the final conformation obtained from the sMD simulations conducted for the glycosylation step. The resulting intermediate structure was an oxazoline. The Glc-oxazoline (Oxa) unit was parametrized using the antechamber module with the GAFF2 ^69^ force field. RESP^70^ charges were calculated at the HF/6-31G* level of theory using Gaussian 09.^71^ We removed the leaving group at subsites +1, +2, +3, and +4 from the svHyal active site, and corrected the system using *tleap*. We conducted cMD simulations to allow solvent water molecules to enter the active site, following the same MD protocol as before, but with harmonic constraints to ensure that the positioning of Asp_110_ and Glu_112_ residues remained compatible with the nucleophilic attack by an incoming water molecule. The detailed protocol can be found in Table S3 (Supporting Information—Section II, Methodology).

We performed a clustering analysis using a TCL script and the GROMOS clustering method to identify reactive conformations for the study of the deglycosylation step. The procedure was similar to the one developed for the first step. First, we filtered the trajectory frames to select only those that contained water molecules within 4 Å of both the intermediate’s anomeric carbon and the carboxyl carbon of Glu112. After filtering the trajectory, we conducted RMSD-based clustering to explore the conformational heterogeneity of the selected frames. The most representative structure from the entire trajectory was then selected for the QM/MM sMD simulations.

### QM/MM MD Simulations: Deglycosylation Reaction Step

We proceeded with the deglycosylation step for the Asp-COOH model, performed under the same QM/MM conditions as the first reaction step. We submitted the selected snapshot for sequential minimizations, followed by a 5 ps *NVT* equilibration before the US simulations.

The QM layer contained 95 atoms, a total charge of −1, a singlet spin multiplicity, and unit cell dimensions of 26.26 Å × 23.00 Å × 20.32 Å. It was defined by the Asp110 and Glu112 side chains in their neutral state, Tyr183 and Tyr230 phenol groups, the disaccharide corresponding to subsites −2 (GlcA) and −1 (Oxa), and the nucleophilic water molecule.

We conducted sMD simulations for 5 ps (time step of 1 fs) along the reaction coordinate (RC_2_) defined as CV_2_ = *d*_3_ – *d*_4_. CV_2_ represents breaking the covalent bond between the oxygen and carbon of the oxazoline pyranose ring (*d*_3_ = O Oxa – C Oxa) and the nucleophilic attack of the water molecule on the Oxa anomeric carbon (*d*_4_ = O Wat – C Oxa) (Figure 3). The RC_2_ value was set to increase from an initial distance of −2.63 Å to the desired final distance of 1.7 Å, capturing opposite changes in the distances as the reaction proceeds, with a harmonic force constant of 250.0 kcal mol^–1^ Å^–2^.

### Umbrella Sampling (US) Simulations

The initial structures for the US^72^ simulations were derived from the trajectories generated during both sMD simulations.

For the glycosylation step, we defined 49 and 55 windows spaced 0.1 Å apart along the RC_1_ for the Asp-COO^–^ and Asp-COOH models, respectively. Each umbrella sampling window underwent an equilibration stage of 4 and 8 ps in the Asp–COO– and Asp-COOH models, respectively. This was followed by 12 ps production, using an integration time step of 1 fs. This resulted in a total simulation duration of 16 and 20 ps per window, and 0.784 and 1.1 ns for each potential of mean force (PMF), respectively.

For the deglycosylation step, we defined 47 windows spaced 0.1 Å apart along the Asp-COOH RC_2_. Each window underwent 20 ps of MD, with the first 4 ps discarded as equilibration.

To calculate the PMF along RC_1_ and RC_2_, we employed a dump frequency of 5.0 fs to retrieve the CV values for each window. To create the free energy profiles, we used the weighted histogram analysis method (WHAM)^73^ and conducted a Bayesian bootstrap analysis, performing 100 bootstraps to estimate the associated error. We applied a convergence tolerance of 0.0001 kcal·mol^–1^ for CV_1_ and 0.001 kcal·mol^–1^ for CV_2_ (Figures S7 and S8) and set the bins to triple the independent simulated windows. To assess the convergence of the profiles, we collected the umbrella sampling data and calculated the PMF over blocks of 2 ps. We confirmed convergence when the recalculated profiles overlapped, indicating that the results had converged (Figures S9–S13).

## Results and Discussion

### Induced-Fit Binding and Catalytic Preorganization of Hyaluronic Acid by svHyal

A detailed qualitative and quantitative analysis of the dynamic interactions between svHyal and its substrate can be found in the Supporting Information, Section III – Results and Discussion, Figures S14–S18. We analyzed the structural dynamics of the hyaluronic acid substrate bound to the svHyal active site before the start of the reaction cycle. After the docking of the substrate, we observed a puckering of the substrate pyranose ring of the GlcNAc unit at subsite −1 (Figure 4), i.e., the GlcNAc residue changed from its ground-state chair conformation (^4^C_1_) toward a distorted boat/skew-boat conformation (^1,4^B/^1^S_3_) (Figure 4), according to the calculated values of the Cremer-Pople puckering angles^74^ (Table S4). Pucker conformation was maintained throughout the MD simulation, likely due to strong stabilization. However, it is noteworthy that standard simulation time scales and the GLYCAM force field may prevent the transition to alternative conformations, limiting the capture of less-populated states.^75,76^ The binding of hyaluronic acid induced a slight closure of the loops surrounding the active site into a “sandwich-like” conformation (Figure 4), a structural rearrangement commonly observed in GH enzymes.^77,78^ Electrostatic, hydrophobic, and stacking contacts involving mostly tyrosine residues are maintained in more than 70% of the simulation time (Figures 4 and S18), highlighting their functional importance in substrate binding and stabilization. Contacts spanning subsites −4 to −2 are predominantly hydrophobic (Leu47, Tyr56, Met308), with Asn58 and Ser307 contributing through polar interactions. Moreover, tyrosine (Tyr183, Tyr191, Tyr230, and Tyr277) and tryptophan (Trp122 and Trp305) residues form a tightly packed pocket that accommodates both the GlcNAc and GlcA residues at subsites −1 to +1. This pocket is thought to shield the intermediate of the reaction from the solvent, contributing to the stabilization of the transition state.^31^ Within this pocket, Asp110 and Tyr230, which interact with the GlcNac unit at subsite −1 throughout the simulation (Figure 4, right), are known to play an important role in positioning the 2-acetamido carbonyl oxygen for nucleophilic attack at the anomeric center.^29^ Finally, contacts spanning subsites +2 to +4 are primarily mediated by the side chains of arginine and lysine residues (Arg115, Lys125, and Lys194), which interact with hyaluronic acid’s negatively charged carboxylic groups.

**Figure 4:**
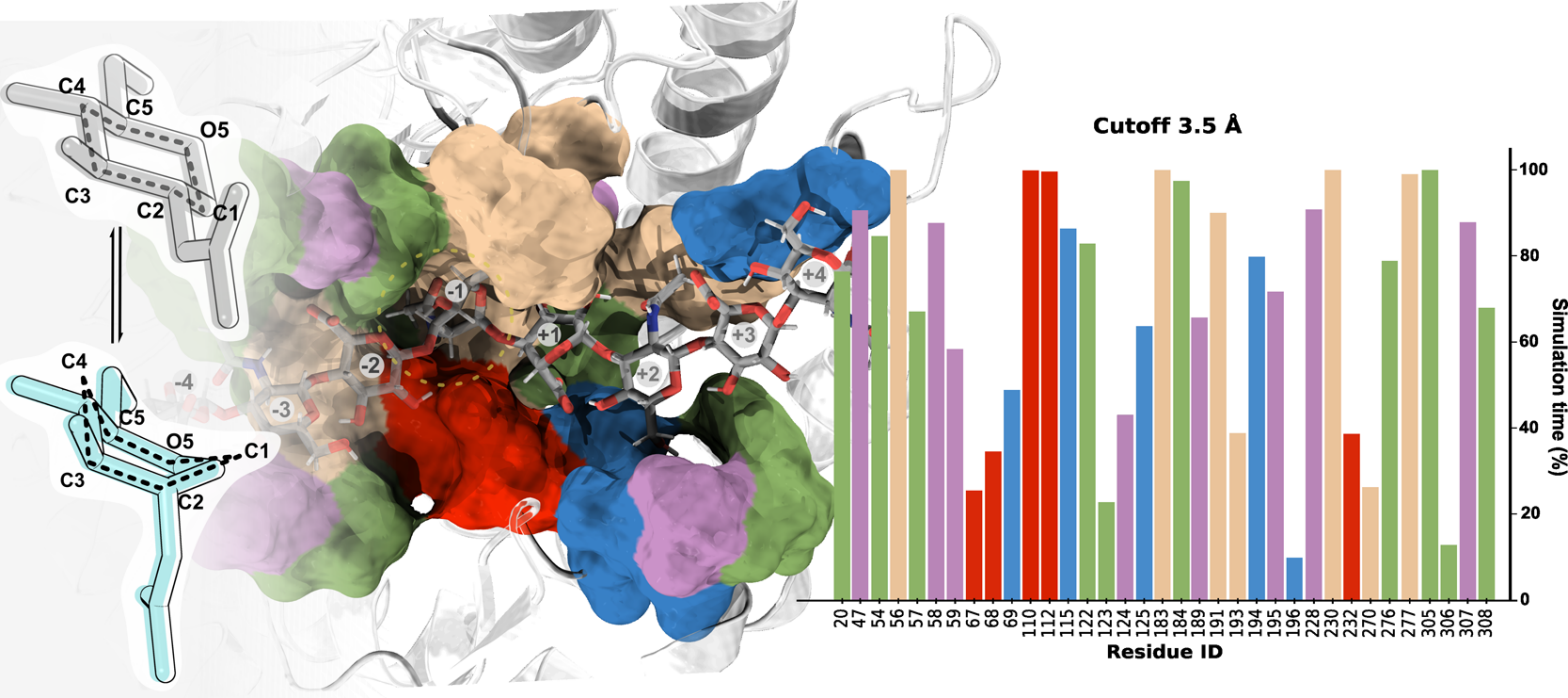
Ring distortion, binding pocket composition, and interaction frequency. (Left) Chair (^4^C_1_) and boat (^1,4^B/^1^S_3_) conformations of the pyranose ring at subsite −1 in the unbound (gray) and bound (light blue) form; The hyaluronic acid-binding pocket is shown with residues colored by physicochemical properties: hydrophobic (green), positively charged (red), negatively charged (blue), polar uncharged (pink), and tyrosines (yellow). The octasaccharide substrate spanning subsites −4 to +4 is displayed in its most prevalent position, observed in 72% of the simulation time across concatenated trajectories. (Right) A histogram displaying the percentage of configurations in which the pocket residues establish contacts with the substrate within 3.5 Å. Only residues involved in contacts maintained for more than 10% of the simulation are shown.

Therefore, both the change in ring puckering and the binding interactions indicate an induced-fit type of mechanism that facilitates substrate-assisted catalysis, in line with a previous proposal for bvHyal-1.^21^ This indicates that svHyal structurally preorganizes the substrate for the first step of the catalytic process through steric deformations and intermolecular interactions within the active site (Figure 4).^41,79–81^ These findings are also in good agreement with those reported by Davies et al.,.^82^

Finally, to determine the precise position of the hyaluronic acid in the svHyal-1 active site, we employed a clustering approach to assess the most prevalent conformations in both models. Our analysis indicated that the conformation in which the substrate is extended along the hyaluronic acid-binding site groove with a slight bending toward the solvent is predominant (72% of the trajectory) (Figure 4, left). However, visualizing the trajectory, we often noticed misalignment of catalytic residues. For instance, when the hyaluronic acid substrate adopted an almost fully extended conformation, the carboxylate oxygen of the assisting Asp110 was often improperly oriented relative to the 2-acetamido group, or the glycosidic oxygen relative to the catalytic Glu112 residue. Such misalignments preclude proton transfer, a crucial step in the reaction mechanism.

### Defining Catalytically Productive Conformations

To identify catalytically competent conformations for subsequent mechanistic studies, we filtered the MD trajectories based on the interatomic distances more directly involved in the chemical reaction. Specifically, we considered the Oε2 Glu112–O4_glyc_ ≤ 4.0 Å and Oδ1 –H2N NAc ≤ 3.0 Å distances to ensure that the catalytic Glu112 remained positioned for proton donation, while Asp110 retained its role as a stabilizer of the positive charge that should develop at the hyaluronic acid nitrogen during the reaction.

As shown in Figure 5, 49% and 38% of the trajectories fall within these criteria in the Asp-COO^–^ and Asp-COOH models, respectively, satisfying distance thresholds that facilitate catalysis.

**Figure 5:**
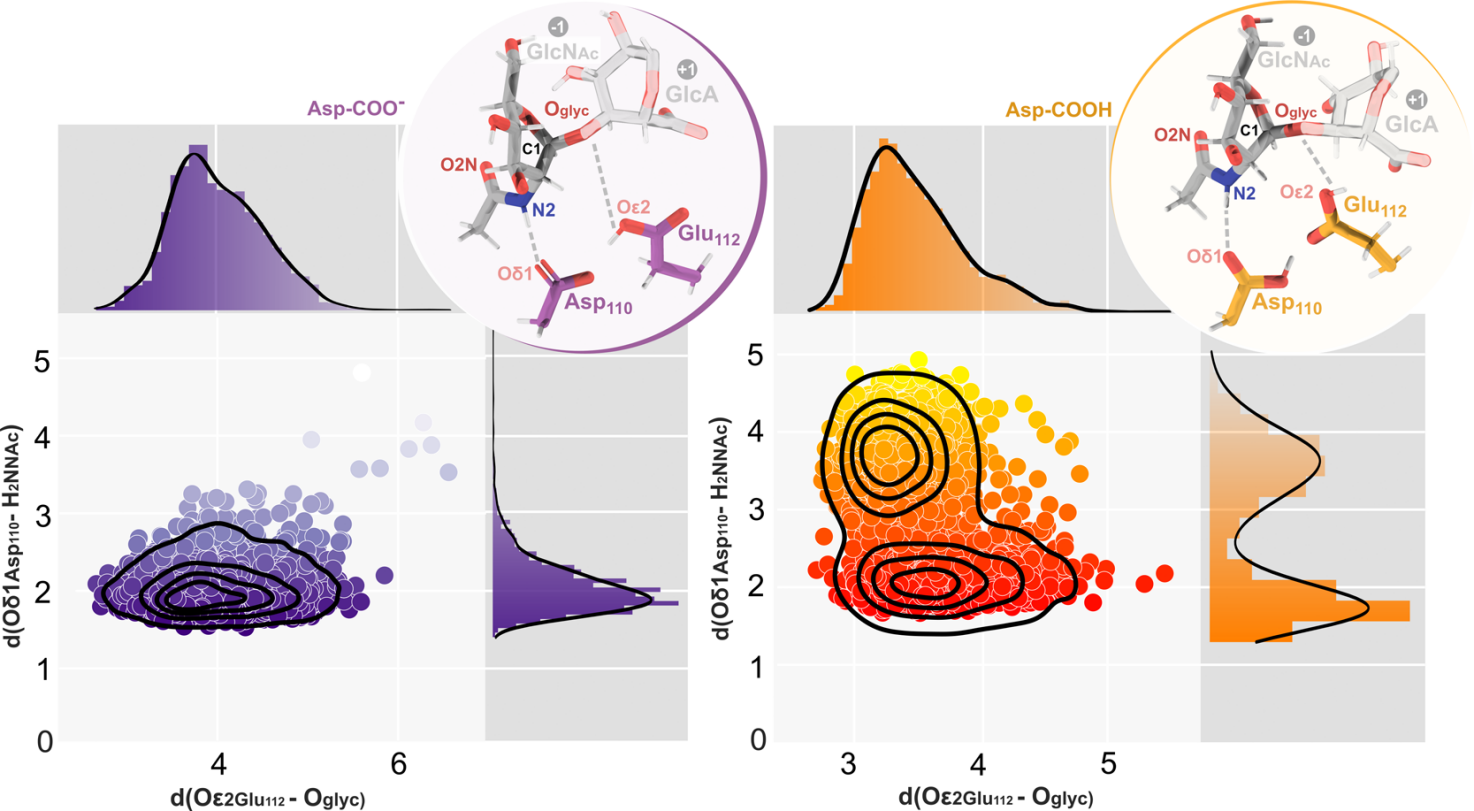
Distance-based clustering. Distribution of the Asp-COO^–^ (Left) and Asp-COOH (Right) trajectory along the Oε2 Glu112–O_glyc_ and Oδ1 Asp110–H2N GlcNAc distances. Darker colors indicate better preorganized conformations for the reaction mechanism. The Michaelis–Menten complex for each model is also represented in circled boxes.

Next, we performed RMSD-based conformational clustering. Figure 5 shows the centroid of the most populated cluster and the selected Michaelis complex for both the Asp-COO^–^ and Asp-COOH models.

The assisting Asp110 and the acid/base Glu112 residues in the Michaelis complex are linked by a hydrogen bond in both models. As in other GHs, the reaction pathway starts with the GlcNAc pyranose ring in the induced-fit boat/skew-boat (^1^S_3_/^1,4^B) conformation (φ = 229.38 ± 8.23, θ = 83.69 ± 1.89), rather than the aqueous solution ground-state ^4^C_1_-chair conformation.^83–87^ Accordingly, the boat/skew-boat conformation facilitates Glu112 proton transfer by placing it between 3.00 and 4.00 Å away from the equatorial glycosidic oxygen (O_glyc_) and enhances the nucleophilic character of the 2-acetamido oxygen (O2).^79^ No other GlcNAc residues of the substrate exhibit this shift between chair and boat conformations, favoring the lower energy ^4^C_1_-chair conformation.

This induced-shift mechanism, in which only the ring involved in catalysis undergoes distortion, is a notable example of how natural evolutionary pressures can invert its relative stability in comparison to its isolated form in solution. In fact, studies have demonstrated that GHs use this strategy to preactivate substrates for efficient catalysis, effectively reducing the activation free energy barrier by 6–10 kcal·mol-1.^80,88^

With the Michaelis complex defined and catalytically productive poses identified, we next examined the glycosylation step using US simulations.

### The Glycosylation Step

The US simulations conducted along the selected coordinate that combines the distances involved in the nucleophilic attack (O2N GlcNAc–C1_anom_) and proton transfer (Oε2 Glu112–O_glyc_), indicate a stepwise dissociative reaction. This is evident from the identification of three minima at RS (resting state), REACT (reactants), and INT1 (reaction intermediate), which are separated by two maxima at TS_RS and TS1 (transition state) (Figure 6).

**Figure 6:**
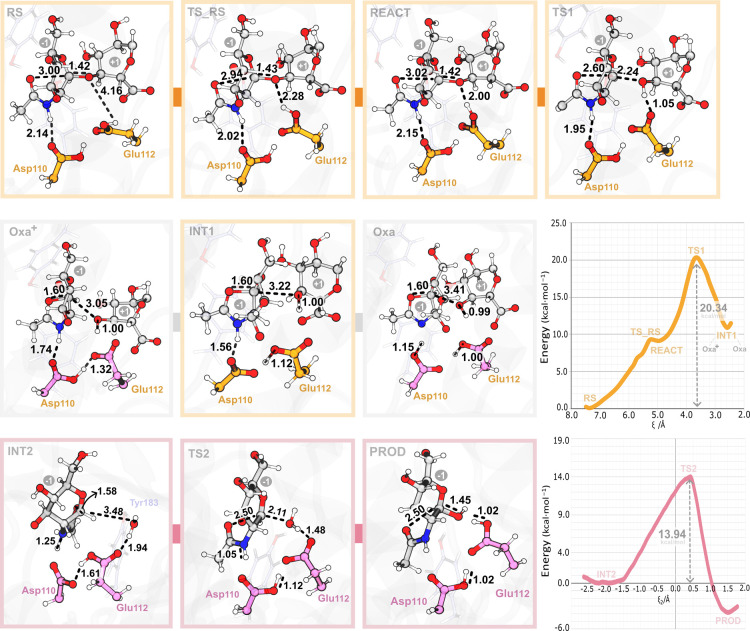
Stick representation of the representative structures corresponding to different stationary points along the glycosylation (orange) and deglycosylation (pink) steps in the Asp-COOH model. The nonstationary states Oxa^+^ and Oxa, which precede and follow the reaction intermediate INT1, are also illustrated. The PMFs are represented by bold orange (deglycosylation) and bold pink (deglycosylation) lines.

From RS to REACT, both models undergo a structural rearrangement of the catalytic proton donor (Hε2 Glu112), optimizing its orientation toward the glycosidic oxygen (O4_glyc_) atom. As a result, the Hε2 Glu112–O4_glyc_ distance decreases from ≈4 to ≈3 Å, in both models, while the carboxyl group of Glu112 rotates from an *anti* to *syn* conformation relative to the glycosidic bond, as observed in other studies related to the GH family.^34,89,90^ The energy required for this process was ≈17 kcal·mol^–1^ and ≈9 kcal·mol^–1^ in the Asp–COO– and Asp-COOH models, respectively. Figure 6 shows the PMF and the most relevant structures along the reaction pathway for the Asp-COOH model, which is the only one kinetically viable, as will be shown below. The equivalent data for the Asp-COO^–^ model is shown in Figures S19 and S20, and its distance values are reported in Table S5. In the Asp-COO^–^ model, Glu112 is hydrogen-bonded to Asp110 but breaks this bond after the initial rearrangement. In contrast, in the Asp-COOH model, the catalytic Glu112 stays hydrogen-bonded with Asp110 (2.31 Å), which, in turn, is hydrogen-bonded to the amide nitrogen of the 2-acetamido group (2.15 Å). As the catalytic Glu112 approaches the oxygen of the glycosidic bond to a distance of ≈2.00 Å (H Glu112–O4_glyc_), the carbonyl oxygen of the GlcNAc initiates an intramolecular nucleophilic attack at the anomeric carbon (C1_anom_–O2 NAc) (Figure S20).

At the transition state of the nucleophilic attack in Asp-COOH, the O_glyc_–H Glu112 and C1_anom_–O2 NAc distances further decrease to 1.05 and 2.60 Å, respectively, while the C1_anom_–O4_glyc_ distance increases from 1.42 to 2.24 Å. Consequently, an oxocarbenium ion-like TS1 (Figure 6—TS1) is formed where the C1_anom_–O5 bond shortens from 1.47 to 1.31 Å (Figure S21 and Table S6), gaining a significant double bond character. Hence, the hybridization of the anomeric center (C1_anom_) changes from sp^3^ to sp^2,^ and delocalization of the positive charge onto the ring oxygen (O5) occurs (from −0.54 e to −0.36 e) (Table S7).

As evidenced by the evolution of the distances along the reaction path in Table S5 and Figure S20, once the protonation of the glycosidic oxygen occurs, the nucleophilic attack and cleavage of the glycosidic bond proceed synchronously.

The computed activation free energy barrier (Δ*G* ^⧧^) for the glycosylation step of the Asp-COO^–^ model is 34.48 kcal·mol^–1^ (Figure S19), in contrast to the Asp-COOH model, which presents a nearly half-lower barrier of 20.34 kcal·mol^–1^. The latter value aligns well with experimental and computational data obtained for other GH-related reaction mechanisms (*k*_cat_ = 28 s^–1^, Δ*G* ^⧧^ ≈ 16 kcal·mol^–1^).^41^ Due to the significant difference between the activation free energy barriers and, principally, the very high barrier of the Asp-COO^–^ mechanism, the Asp-COOH is dominant and the only one mechanistically feasible.

Throughout the reaction, from the TS1 to INT1, the H Glu112–O4_glyc_ and C1_anom_–O2 NAc distances further decrease to 1.00 Å and 1.60 Å, respectively, while the glycosidic bond stretches to 3.22 Å, breaking up completely. Consequently, a covalent bond is formed between C1_anom_ and O2 NAc. The N-acetyl group then polarized, and a partial positive charge develops (from −0.55 to −0.47e) on the amide nitrogen (N2) atom (Table S7).

This leads to the formation of a bicyclic oxazoline (Oxa) intermediate in the Asp–COO^–^ model and a bicyclic Asp_110_-stabilized oxazolinium ion (Oxa^+^) intermediate in the Asp-COOH (Figure 6—INT1). The latter represents a structure that lies between two other possible intermediate species, an oxazolinium ion (Oxa^+^), and an oxazoline (Oxa) (Figure 6—Oxa^+^ and Oxa). It is characterized by a first proton transfer from Asp110 to the deprotonated Glu112, preceding a second proton transfer from the Oxa^+^ nitrogen atom toward Asp110 in an almost barrierless manner (≈0.90 kcal·mol^–1^). These findings align with those previously described for Chitinase A and Chitinase B GH 18 enzymes.^41,42^

The reaction free energy (Δ*G*_r_) associated with the formation of the reaction intermediate was 16.22 kcal·mol^–1^ for the Asp–COO^–^ model and 10.80 kcal·mol^–1^ for the Asp-COOH model, suggesting an endergonic reaction in either case. The endergonic character of the glycosylation step also agrees with previous studies.^37,42,91^

### The Deglycosylation Step

The last snapshot of the sMD trajectory, corresponding to the oxazoline species, was used for subsequent MD simulations, which were performed to allow water molecules to enter and occupy the active-site space left available after removal of the leaving group produced from the prior hydrolysis (Figure S22). A representative frame from this MD simulation was then retrieved as the starting structure for the second and final step of the double displacement substrate-assisted mechanism. Only the Asp-COOH model was studied from this point onward because the barriers found in the Asp-COO^–^ model during the glycosylation step were prohibitively high. The starting structure contained a water molecule originating from the bulk solvent and positioned at 4.04 Å from C1_anom_, forming hydrogen bonds with Glu112 (2.06 Å) and Tyr183 (1.78 Å). It is noteworthy that, in some retaining GHs (GH33 and GH34), this tyrosine can act as a nucleophile during the glycosylation step, functionally equivalent to the carboxylate of classical retaining GHs^92^ However, in the svHyal case, it is unlikely that Tyr183 played this role, since it would require a substantial reduction in its p*K*_a_, and there is no charged residue nearby to assist with this function. Thus, the role of Tyr183 appeared to be to ensure that the water molecule is properly oriented for the catalytic deglycosylation process. The active site residues Asp110 and Glu112 maintained their network of hydrogen-bond interactions necessary for a productive reaction.

The deglycosylation reaction was simulated by biasing the nucleophilic attack of the water molecule on the anomeric center (O_Wat_–C1_anom_) and concomitant breaking of the bond between the Oxa oxygen and the anomeric carbon (O2 Oxa–C1_anom_). As evidenced by the resulting PMF (Figure 6), a transition from oxazoline to oxazolinium ion-like (i.e., a proton transfer from the Oδ2 Asp110 atom to the N2 Oxa atom) occurs almost immediately, with an associated barrier of 1.11 kcal·mol^–1^ (Figure 6). This suggests that the oxazolinium ion-like intermediate is slightly more energetically favorable than the oxazoline species within the Asp-COOH model.

To further clarify whether the intermediate was an Glc-oxazolinium ion (Oxa^+^) or an oxazoline (Oxa), we ran a 30 ps unbiased NVT QM/MM MD simulation. The Asp110 residue remained neutral in only 30.6% of the simulation time, with frequent interconversion between Oxa and Oxa^+^ occurring after ∼8 ps. The Oxa^+^ conformer was dominant (69.4%), indicating that it is slightly more stable, while Oxa is a transient species. These results further corroborate a very small free energy difference between the two states, consistent with the behavior described by Iino et al.,^42^ for Chitinase A. The interconversion proceeds via a proton relay mechanism^42^ involving Asp110 and Glu112, with proton transfers regenerating the Oxa^+^ species (Figure 6—INT2).

Hydrogen bonding between Tyr230 and the 2-acetamido group carbonyl oxygen may also destabilize the reaction intermediate (Oxa^+^), promoting the transfer of the Oxa^+^ proton to Oδ2 Asp110. In the Oxa species, the resonance within the amide group delocalizes electron density, making the carbonyl oxygen have a partial negative character. The evolution of distances during the deglycosylation steps is presented in Table S8 and Figure S23.

According to the US simulations, the deglycosylation step is initiated by the approach of the nucleophilic water molecule to the Oxa^+^ anomeric center. This triggers the transfer of a proton from the water molecule to Glu112, as the H Wat–O Glu112 distance decreases from 1.94 to 1.48 Å. The transition state (TS2) forms during the general base-catalyzed attack of the water at the C1_anom_ (Figure 6—TS2). Consequently, the O2N Oxa^+^–C1_anom_ distance elongates from 1.58 to 2.50 Å.

The free energy barrier for the deglycosylation step is 13.94 kcal·mol^–1^, consistent with experimental data and computational studies on other GH enzymes (11.6 kcal·mol^–1^).^93–95^ As the reaction progresses to the products (Figure 6—PROD), the catalytic Glu112 fully deprotonates the water molecule, and the latter forms a covalent bond with C1_anom_, weakening the O2N Oxa^+^–C1_anom_ bond and restoring the GlcNAc unit. In addition, the strong hydrogen bond between the 2-acetamido carbonyl group and the Tyr230 hydroxyl group is regenerated (distance decreased from 2.40 Å at INT2 to 1.77 Å at PROD), supporting its role as a stabilizer of the substrate, as discussed in previous investigations.^37,41,96^ The reaction free energy associated with this step was −3.87 kcal·mol^–1^, indicating an exergonic reaction.

### Mapping the Puckering Itinerary during SvHyal Catalysis

Understanding the conformational changes that the hyaluronic acid GlcNAc unit at subsite −1 undergoes during the reaction is relevant for the design of potent inhibitors.^82,97^ To investigate these changes, we examined the substrate puckering itinerary along the reaction pathway. We used the classical puckering parameters (Q, θ, and ϕ), which were projected onto Stoddard’s pseudorotational diagram.^98,99^

The analysis of the Cremer-Pople puckering angles of the substrate pyranose ring at subsite −1 revealed a ^1,4^B/^1^S_3_ → [^4^E/^4^H_3_]^⧧^ → ^4^C_1_ conformational itinerary along the glycosylation step (Figure 7 and Table S9), consistent with predictions for other retaining GHs.^34,79,82,85,100^ In the reactant state, the pyranose ring of the GlcNAc unit adopts an intermediate conformation between ^1,4^B (boat) and ^1^S_3_ (skew-boat), in both Asp-COO^–^ and Asp-COOH models. This enzyme-induced conformation preorganizes the pyranose ring for the first reaction step, as it closely resembles the geometry of the TS, lowering the free energy barrier to reach it. Additionally, the ^1,4^B/^1^S_3_ conformation enables an optimal orientation of the catalytic residues (Asp110 and Glu112). This geometry requires less energy for distortion in comparison to other puckering conformations.^79,82,101,102^

**Figure 7:**
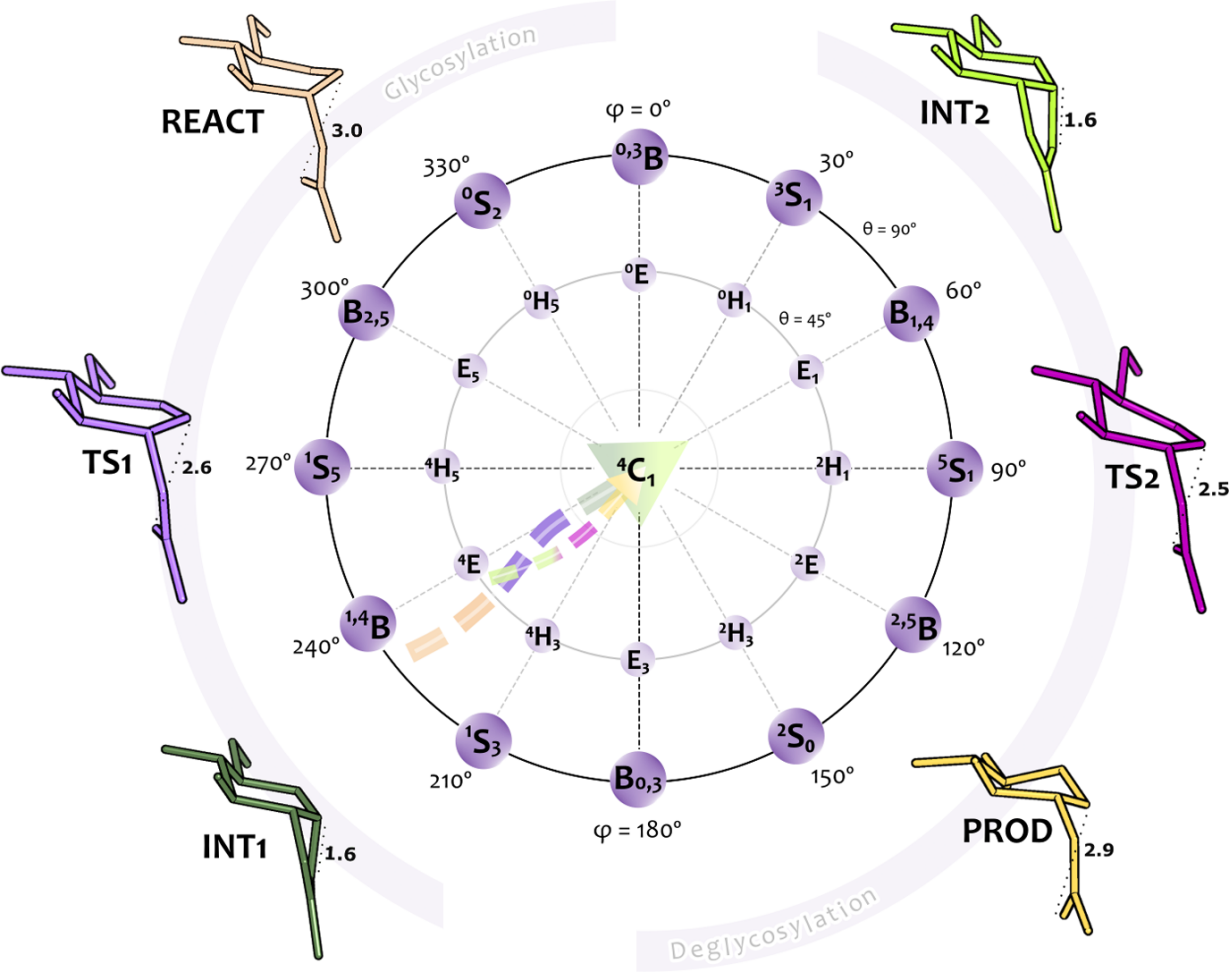
Stoddart’s pseudo rotational diagram (centered on the ^4^C_1_ conformation) with a top-down view of the northern hemisphere of the Cremer–Pople puckering sphere. The diagram shows the puckering conformations of the pyranose ring at subsite −1 for the different stationary points along the glycosylation (thicker arrow) and deglycosylation (thinner arrow) reaction pathways. The colored arrows represent the obtained itineraries for both steps; each color corresponds to the puckering conformation of each stationary point (REACT—beige; TS1—violet; INT1—dark green; INT2—light green; TS2—magenta and PROD—yellow).

At the transition state, the Glu112 proton was transferred to the glycosidic oxygen, the glycosidic bond began to cleave, and the nucleophilic attack at the anomeric carbon was underway. As a result, the pyranose ring developed a partial positive charge at the anomeric carbon, leading to delocalization of the lone pair electrons across the sp^2^-hybridized O5–C1 bond (Tables S6 and S7).^103^ Consequently, the pyranose ring C5–O5–C1–C2 atoms were forced into a coplanar arrangement resembling a conformation between the envelope and half-chair ([^4^E/^4^H_3_]^⧧^). This conformation satisfied the stereoelectronic conditions of an oxocarbenium ion-like transition state^34,82,103^ and is among the possible transition-state conformations (^4^H_3_, ^3^H_4_, ^2,5^B, B_2,5_) reported by Davies et al.,^101^ for GHs.

Throughout the final stage of the glycosylation step, the pyranose ring of the Oxa^+^/Oxa intermediate species adopted a distorted ^4^C_1_-chair conformation suitable for subsequent nucleophilic attack by water.^31,33^ It is noteworthy that the precise catalytic itinerary did not follow a radial straight line on Stoddard’s diagram but instead traced a more nonlinear path (Figure 7). Moreover, while the other sugars remained in the lower-energy ^4^C_1_ chair conformation, subunits +1 and +3 adopted a skew-boat (^2^S_o_) conformation. This was probably due to binding constraints imposed by Hyal’s binding pocket, which ultimately may have influenced the endergonic character of the reaction.

Finally, during the deglycosylation step, the pyranose ring at subsite −1 followed a ^4^C_1_ → [^4^E/^4^H_3_]^⧧^ → ^4^C_1_ itinerary, and the stereochemistry at C1 remained unchanged, confirming that the nucleophilic attack of the water molecule at the anomeric center resulted in an overall retention of the anomeric configuration.

### Insights into the Molecular Basis of SvHyal Inhibitor Recognition

Understanding enzyme reaction mechanisms is critical for the rational design of potent inhibitors. The transition state structure serves as a template for designing TS analogues. A study conducted by Whitworth et al.^104^ demonstrated that NAG-thiazoline—a highly potent competitive inhibitor of both β-HexNAcase (GH 20)^34,105^ and O-GlcNAcases (GH 84)^85,106^ —functions as an optimal TS analogue. We superimposed NAG-thiazoline onto the hyaluronic acid GlcNAc unit and observed that it more closely resembled the oxazoline/oxazolinium ion intermediate conformation than the TS.^34,105^ A similar conformational preference was observed when we superimposed the Gal-thiazoline complex^39^ onto the svHyal active site (Figure 8).

**Figure 8:**
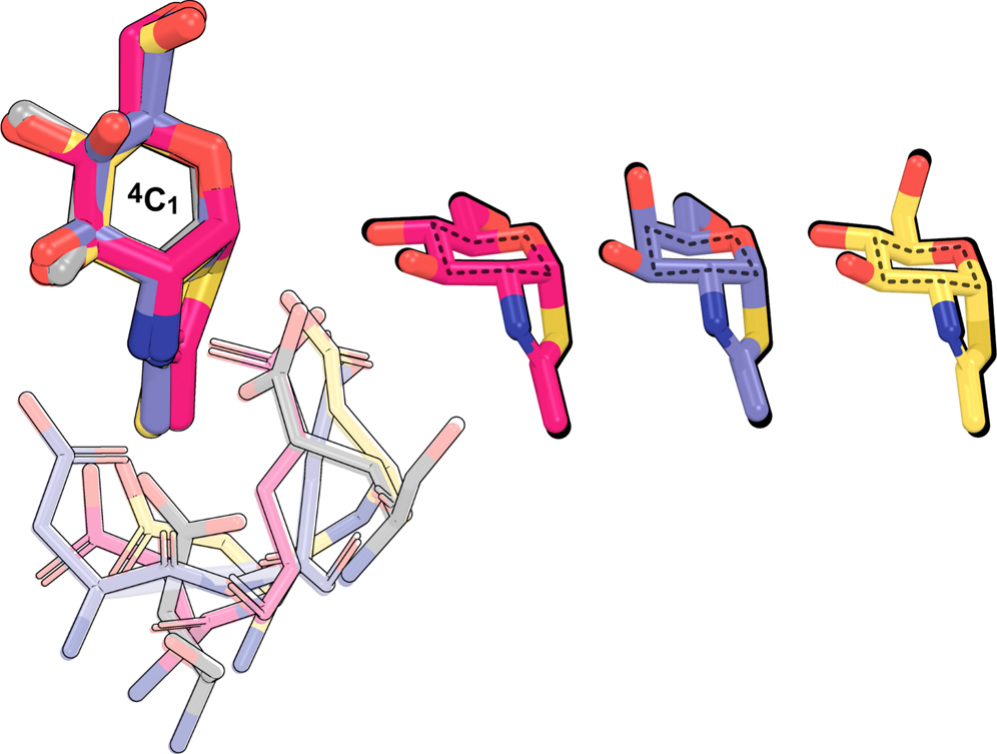
Superimposition of the svHyal oxazolinium ion intermediate (gray) with NAG-thiazoline complexed with β-HexNAcase from *Paenibacillus* sp. (magenta, PDB ID: 3SUR), NAG-thiazoline complexed with O-GlcNAcase from *B. thetaiotaomicron* (slate blue, PDB ID: 2CHN), and Gal-thiazoline complexed with BvGH123 from *P. vulgatus* (yellow, PDB ID: 5L7V). Dashed lines illustrate the ^4^C_1_-chair conformation adopted by the reaction intermediate. Thinner sticks show the similar positioning of the catalytic residues across the different GH families.

This was previously observed by Whitworth et al.,^104^ because these inhibitors, with their anomeric carbon positioned below the ring plane, are similar to the ^4^C_1_ conformation adopted by the oxazoline/oxazolinium ion intermediate, consistent with the conformational itinerary obtained for the glycosylation reaction. Despite not being perfect TS mimics, the oxazoline and oxazolinium ion intermediate analogues are closely related to the TS and have been used as scaffolds for GH inhibitor design.^31,34,80,85,105,106^ The TS corresponds to an oxocarbenium ion-like species with partially formed (O2N NAc–C1 NAc)/broken (C1_anom_–O4_glyc_) bonds, which is extremely unstable and short-lived. By contrast, the oxazoline/oxazolinium ion intermediate species, with their cyclized ring, are much more stable. This relative stability facilitates the design of drug analogues that mimic these intermediate species. In addition, the oxazoline/oxazolinium ion intermediates are still high in energy relative to the reactants, suggesting that the enzyme might have evolved to stabilize them. The strong enzyme–intermediate interactions may help explain the high affinity observed for such analogues.

Interestingly, GH families that follow the substrate-assisted retaining mechanism, including svHyal, display a remarkably similar spatial arrangement of the catalytic residues, despite showing little to no conservation in their sequences^39^ (Figure 8). These findings emphasize that mimicking the TS conformation is not the only approach to achieve potent inhibitors. Although the common strategy for the design of inhibitors involves mimicking the TS chemical features, the example of NAG-thiazoline illustrates that intermediate-like structures with both similar geometry and electrostatic distribution can also serve as high-affinity intermediate analogues.^97^ Thus, based on these examples, it is important to incorporate specific intermediate features, such as geometry and charge, in the design of potent svHyal inhibitors.

## Conclusions

We studied the reaction of hyaluronic acid hydrolysis catalyzed by hyaluronidase from the *B. arietans* viper venom, revealing a stepwise retaining mechanism. We investigated substrate conformational changes within the svHyal active site, and our findings support an induced-fit mechanism. Intermolecular interactions at subsites −1, +1, and +2 and the intrinsic chemistry of the GlcNAc unit at subsite −1, preactivate hyaluronic acid for reaction by inducing a conformational change from the ^4^C_1_ chair (unbound) to the higher-energy ^1,4^B/^1^S_3_. This distortion toward a high-energy, transition-state-like conformation, combined with the precise alignment of the Asp110 and Glu112 catalytic residues, provides the stereoelectronic criteria necessary for efficient catalysis. Although the energetic cost of these events is considerable (9 kcal·mol^–1^), it is compensated by a subsequent reduction in the free energy barrier.

SvHyal employs a proton shuttle mechanism that plays a crucial role in the degradation of hyaluronic acid. During the glycosylation step, the protonation of the glycosidic bond and the nucleophilic attack at the anomeric center initiate a cascade of proton-transfer events involving Asp110, Glu112, and the substrate. Modeling Asp110 as protonated, the glycosylation reaction step resulted in an activation free energy of 20.34 kcal·mol^1^, nearly half the barrier of the negatively charged Asp110 pathway. This suggests that the neutral Asp110 protonation state favors the lowest energy pathway and helps stabilize the reaction intermediate.

A neutral oxazoline species forms transiently through rapid interconversion, facilitated by the proton relay-like pathway. These findings underscore the critical role of this hydrogen-bond network in fine-tuning the enzymatic reaction of the svHyal.^41,45,80,107^

The deglycosylation step, triggered by the approach of a nucleophilic water molecule to the anomeric center, proceeds via a mirrored sequence of proton-transfer steps involving the same residues, highlighting the reversible character of the svHyal reaction mechanism. This second step of the double-displacement substrate-assisted reaction yields an activation free energy of 13.94 kcal·mol^–1^, confirming the glycosylation step as the rate-limiting step.^37,91^

In summary, this study highlights the importance of accurately defining the chemical mechanism of hyaluronic acid degradation by svHyal and the svHyal-induced distortions and sugar ring puckering angles. SvHyals appear to have evolved to preferentially recognize and stabilize certain ring conformations that are similar to the TS structure, thereby facilitating charge delocalization and optimizing catalysis. These processes must be considered when designing conformation-specific inhibitors to neutralize svHyals spreading activity, which exacerbates the deadly effects of snakebite envenomation.

Our study clarifies the importance of Hyal enzymes, supporting the increasing interest in them. By regulating the breakdown of hyaluronic acid, Hyal inhibition provides a broad therapeutic and cosmetic potential, which includes reducing inflammation, slowing aging, and supporting a variety of medicinal and biotechnological uses.

## Supplementary Material





## Data Availability

The data supporting this article have been included as part of the Supporting Information.

## Notes

Parameters and coordinate files of the initial system (ZIP)
Chemical structure of hyaluronic acid; Schematic representation of hyaluronic acid degradation by Hyal; Interspecific conservation among Hyal homologues; Classical double-displacement reaction mechanism; p*K*_a_ Values; Molecular docking and binding analysis; Molecular dynamics simulation setup and parameters; Convergence and density distributions profiles for US; PMF calculations over 2 ps time blocks; MD Analysis: distances, RMSD, RMSF, contacts, and pucker angles; PMF profile of the Asp-COO^–^ reaction mechanism and stationary state structures; Geometric and charge analysis along the reaction pathway; Radial distribution of water molecules in the active site after intermediate formation­(PDF)
