# Supplementary material for: Mechanism of Hyaluronic Acid Hydrolysis Catalyzed by Snake Venom Hyaluronidase
Source: J Chem Inf Model. 2026 Jan 27;66(7):4033–48. doi: 10.1021/acs.jcim.5c02594 (PMC13080975; doi:10.1021/acs.jcim.5c02594)
Supplement: Supplementary file 2 [file ci5c02594_si_002.pdf]

Supporting Information

Mechanism of Hyaluronic Acid Hydrolysis Catalyzed by Snake Venom Hyaluronidase

Juliana Castro-Amorim, Maria J. Ramos, Pedro A. Fernandes*

LAQV, REQUIMTE, Departamento de Química e Bioquímica, Faculdade de Ciências, Universidade do Porto, Rua do Campo Alegre, s/n, 4169-007 Porto, Portugal

*Email: pafernan@fc.up.pt

This PDF includes:

	Content	Page
**Figure S1**	Chemical structure of hyaluronic acid	S2
**Figure S2**	Hyaluronidase-mediated hyaluronic acid degradation.	S2
**Figure S3**	Comparative structural analysis and sequence identity matrix of hyaluronidases across taxa	S3
**Figure S4**	Schematic diagram of the classical double-displacement mechanism	S3
**Table S1**	pKa values of the hyaluronidase's titratable residues at pH 6	S4
**Method**	Molecular docking and complex formation	S5
**Figure S5**	Representation of the spatial constraints applied during the docking process	S5
**Figure S6**	Pose prediction of the tetrameric product by GOLD's standard scoring functions	S6
**Method**	Classical Molecular Dynamics Simulations	S7
**Table S2-3**	Detailed protocols for molecular dynamics simulations	S7-8
**Figure S7**	Free energy profiles along the first reaction coordinate (RC_1) – Glycosylation	S9
**Figure S8**	Free energy profiles along the second reaction coordinate (RC_2) – Deglycosylation	S9
**Figure S9**	US window density distributions for RC_1 (Asp-COO^- model)	S10
**Figure S10**	US window density distributions for RC_1 (Asp-COOH model)	S10
**Figure S11**	US window density distributions for RC_2 (Asp-COOH model)	S11
**Figure S12**	Evolution of PMFs over blocks of 2 ps, along RC_1 (Asp-COO^- and Asp-COOH models)	S12
**Figure S13**	Evolution of PMFs over blocks of 2 ps, along RC_2 (Asp-COOH model)	S13
**Figure S14**	Qualitative and quantitative analysis of Asp-COO^- MD trajectories	S14
**Figure S15**	Qualitative and quantitative analysis of Asp-COOH MD trajectories	S15
**Figure S16**	Time evolution of catalytic distances across Asp-COO^- MD trajectories	S17
**Figure S17**	Time evolution of catalytic distances across Asp-COOH MD trajectories	S18
**Figure S18**	Percentage of svHyal-hyaluronic acid contacts (3.5 Å cutoff)	S19
**Table S4**	Cremer–Pople puckering parameters for the substrate's −1 subunit in unbound, bound, and MD states from concatenated Asp-COO- and Asp-COOH trajectories.	S19
**Figure S19**	Stationary point structures and PMF for the glycosylation step of the Asp-COO- model	S20
**Table S5**	Average bond-distance changes along glycosylation with standard deviations (both models).	S20
**Figure S20**	Time evolution of atomic distances along glycosylation reaction for both models	S21
**Table S6**	Average sugar-ring distances during glycosylation with standard deviations (Asp-COOH).	S21
**Table S7**	Hirshfield charges distribution along the glycosylation step (Asp-COOH model)	S22
**Figure S21**	Evolution of the GlcNAc C1-O5 distance along the Asp-COOH glycosylation reaction	S22
**Figure S22**	Radial distribution function	S22
**Figure S23**	Time evolution of atomic distances along the deglycosylation reaction (Asp-COOH)	S23
**Table S8**	Average bond-distances during the Asp-COOH deglycosylation with standard deviations	S23
**Table S9**	Cremer–Pople puckering parameters for the −1 subunit of the substrate during glycosylation and deglycosylation steps for Asp-COO^- and Asp-COOH trajectories	S24

Section I – Introduction

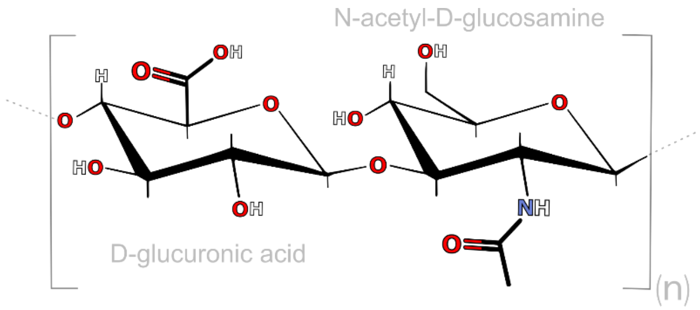

Figure S1: The chemical structure of hyaluronic acid represented by repeating D-guluronic acid and N-acetyl-D-glucosamine units (n).

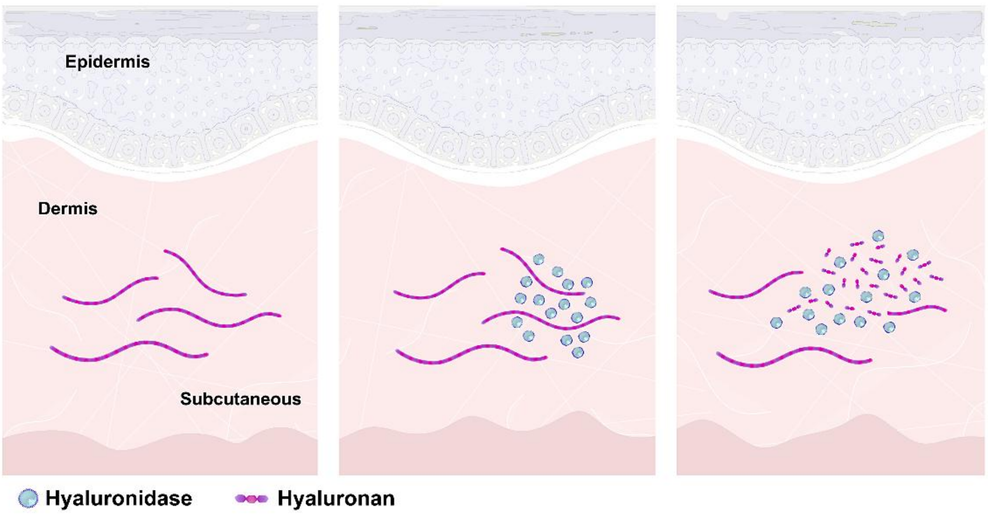

Figure S2: Hyaluronidases (Hyals) catalyze the degradation of hyaluronic acid in the extracellular matrix (ECM), increasing tissue permeability and thus, the adsorption and diffusion of venom components through the tissues.

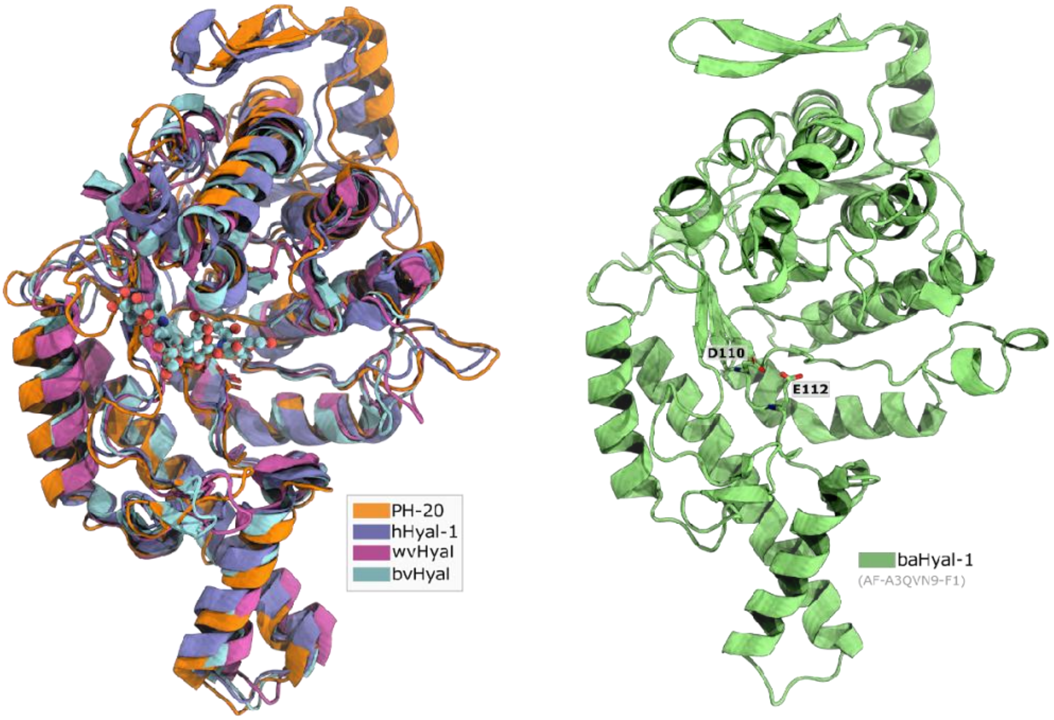

P49370|*Vespula vulgaris*		52.87	33.54	31.17	33.95	33.02	33.95	33.64	33.64
Q08169|*Apis mellifera*	52.87		26.95	29.64	29.17	29.44	29.72	30.28	30.28
P38567|*Homo sapiens*, PH-20	33.54	26.95		40.9	42.06	41.59	42.29	41.36	41.59
Q12794|*Homo sapiens*, Hyal-1	31.17	29.64	40.9		39.86	40.55	40.55	38.94	39.17
J3S820|*Crotalus adamanteus*	33.95	29.17	42.06	39.86		88.42	89.31	88.86	89.09
A3QVN9|*Bitis arietans*	33.02	29.44	41.59	40.55	88.42		93.54	93.1	93.32
A3QVN2|*Echis ocellatus*	33.95	29.72	42.29	40.55	89.31	93.54		94.88	95.1
A3QVN6|*Echis leakeyi*	33.64	30.28	41.36	38.94	88.86	93.1	94.88		99.78
A3QVN3|*Cerastes cerastes*	33.64	30.28	41.59	39.17	89.09	93.32	95.1	99.78	

Figure S3: Comparative structural analysis of hyaluronidases derived across different taxa. **(Top left)** Superimposition of available X-ray crystallography structures illustrating structural conservation among hyaluronidases. Ball-and-stick representation depicts the bvHyal-bound tetramer product from PDB ID 1FCV (blue). **(Top right)** Alphafold model representing the snake venom hyaluronidase for the *Bitis arietans* species (svHyal-1), showcasing its high structural similarity to the previous X-ray structures. **(Bottom)** Sequence identity matrix depicting homology among hyaluronidases from various species, including snakes, humans, wasps, and bees.

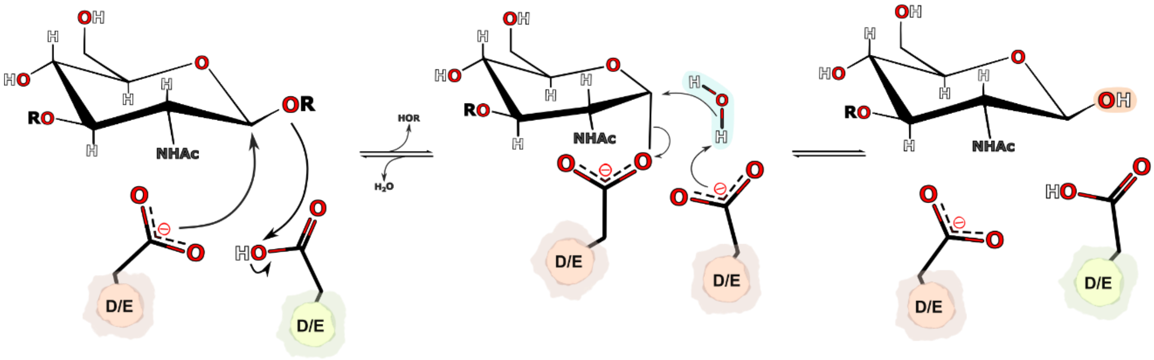

Figure S4: Schematic diagram of the classical double-displacement mechanism with the formation of a glycosyl-enzyme intermediate. An active site carboxylate is the proposed nucleophile in this enzyme-catalyzed reaction. Active site residues can be either Asp or Glu residues, or both.

Section II – Methodology

Table S1: pKa values of the protein titratable residues at pH=6 predicted by PROPKA integrated in the PDB2PQR server. Residues with high sidechain pKa values are highlighted in light red.

Residue	pKa	Residue	pKa	Residue	pKa	Residue	pKa	Residue	pKa
**TYR 8**	11.28	**LYS 4**	8.65	**ARG 27**	12.70	**GLU 11**	4.93	**ASP 31**	3.80
**TYR 28**	10.16	**LYS 29**	10.28	**ARG 69**	12.59	**GLU 68**	4.66	**ASP 33**	2.63
**TYR 56**	12.65	**LYS 35**	10.34	**ARG 96**	13.29	**GLU 81**	4.61	**ASP 45**	5.92
**TYR 63**	16.12	**LYS 85**	10.61	**ARG 115**	12.25	**GLU 112**	6.08	**ASP 67**	1.94
**TYR 128**	12.05	**LYS 89**	10.55	**ARG 120**	11.86	**GLU 138**	3.62	**ASP 93**	2.44
**TYR 156**	14.41	**LYS 91**	10.48	**ARG 129**	11.95	**GLU 142**	3.69	**ASP 110**	8.81
**TYR 179**	12.45	**LYS 101**	9.66	**ARG 131**	12.81	**GLU 145**	4.29	**ASP 119**	2.87
**TYR 182**	14.67	**LYS 125**	8.65	**ARG 150**	12.42	**GLU 155**	5.13	**ASP 146**	3.57
**TYR 183**	15.08	**LYS 137**	10.53	**ARG 165**	13.52	**GLU 157**	5.33	**ASP 166**	4.49
**TYR 185**	12.18	**LYS 147**	10.33	**ARG 175**	11.34	**GLU 172**	5.37	**ASP 87**	3.00
**TYR 191**	13.26	**LYS 149**	10.74	**ARG 211**	13.11	**GLU 173**	4.58	**ASP 192**	4.12
**TYR 193**	12.86	**LYS 153**	11.39	**ARG 220**	13.37	**GLU 208**	3.71	**ASP 198**	3.89
**TYR 200**	13.67	**LYS 154**	10.45	**ARG 236**	13.26	**GLU 232**	4.29	**ASP 206**	3.90
**TYR 230**	18.37	**LYS 158**	10.35	**ARG 248**	14.74	**GLU 251**	4.67	**ASP 213**	3.38
**TYR 263**	11.55	**LYS 161**	9.99	**ARG 254**	14.71	**GLU 261**	4.62	**ASP 221**	3.12
**TYR 270**	15.43	**LYS 194**	11.34	**ARG 260**	12.60	**GLU 280**	4.21	**ASP 239**	3.81
**TYR 275**	11.82	**LYS 196**	10.51	**ARG 272**	12.82	**GLU 285**	4.18	**ASP 262**	3.58
**TYR 277**	10.69	**LYS 203**	10.41	**ARG 330**	12.17	**GLU 293**	3.40	**ASP 286**	4.77
**TYR 310**	12.45	**LYS 243**	10.21	**ARG 348**	12.47	**GLU 369**	4.43	**ASP315**	4.00
**TYR 323**	13.96	**LYS 250**	11.08	**ARG 352**	10.22	**GLU 381**	3.60	**ASP 359**	2.00
**TYR 331**	13.93	**LYS 319**	9.99	**ARG 355**	12.58	**GLU 391**	4.51	**ASP 394**	3.21
**TYR 397**	10.94	**LYS 321**	10.27	**ARG 372**	12.46	**GLU 416**	3.43	**ASP 422**	4.07
**TYR 407**	12.25	**LYS 340**	10.60	**ARG 399**	12.40	**GLU 417**	3.65		
**TYR 414**	10.04	**LYS 349**	10.31	**ARG 424**	12.45				
**TYR 418**	10.40	**LYS 356**	10.17						
**HIS 38**	5.25	**LYS 382**	11.00						
**HIS 59**	5.40	**LYS 383**	11.37						
**HIS 65**	5.21	**LYS 387**	10.32						
**HIS 71**	7.35	**LYS 389**	10.75						
**HIS 74**	5.46	**LYS 393**	10.73						
**HIS 86**	4.02	**LYS 400**	10.40						
**HIS 104**	6.02	**LYS 411**	11.48						
**HIS 140**	6.78	**LYS 421**	10.42						
**HIS 246**	7.08	**LYS 425**	10.54						
**HIS 247**	4.89								
**HIS 344**	5.86								
**HIS 357**	6.26								
**HIS 365**	4.41								
**HIS 376**	6.00								

His71, His140, and His246 were found to have sidechain pKa values higher than those typically observed. However, they formed hydrogen bonds and electrostatic interactions with the surrounding environment.

Molecular Docking and Complex Formation

To predict the binding mode of the octasaccharide substrate within the hyaluronic acid-binding cleft, we employed a multi-docking approach. We used information reported on the crystallized bvHyal-bound tetramer product [21] to aid in the initial docking of the additional segment at subsites +1 to +4. To validate the docking methodology, we re-docked the tetrameric product of the bvHyal-1 crystallographic structure (PDB ID: 1FCV) to the -1 to -4 subsites using the GOLD docking software. The docking box for the target structure was defined using the co-crystallized tetrameric product as the reference ligand, encompassing all space within 10 Å of the ligand atoms. Additionally, 20 docking solutions were generated using GOLD's Genetic Algorithm iterations, and all GOLD's scoring functions were tested, and docking poses were ranked and visually inspected accordingly. We evaluated the docking accuracy and performance by comparing the RMSD values between the docking solutions and the crystallographic pose. Finally, residues identified by *Marković-Housley et. al.* [1], as being involved in interactions, were carefully constrained to ensure that the tetrameric unit was optimally positioned for catalysis (**Figure S5**).

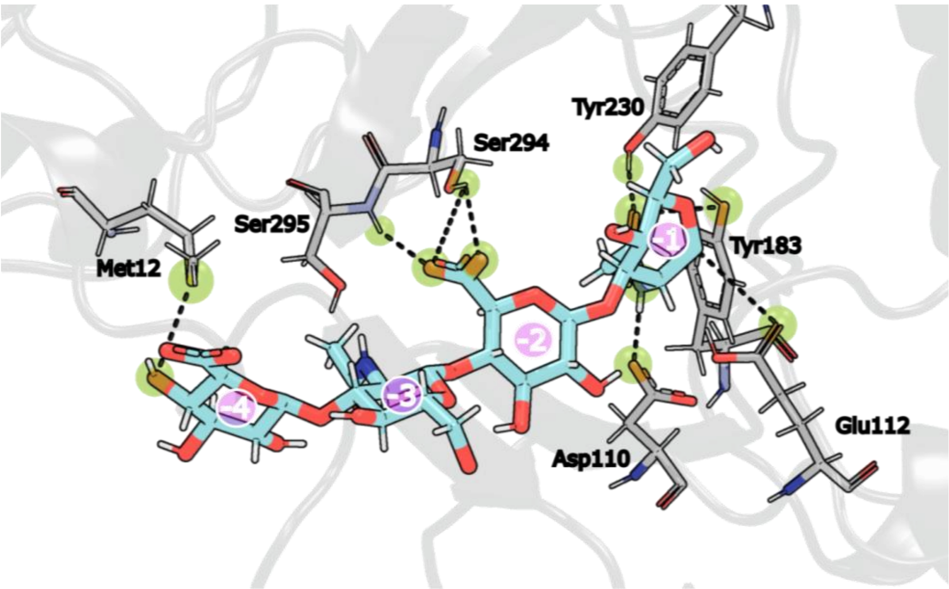

Figure S5: Representation of the spatial constraints applied during the docking process to refine ligand positioning. Green spheres highlight the atoms to which the constraints were applied and dashed lines the interactions.

After analyzing the best-ranked solutions and the RMSDs, we concluded that the ChemScore scoring function yielded the most accurate results. The best-ranked docking solution displayed an RMSD value of 0.92 Å concerning the crystallographic pose. Contacts with svHyal-1 were mainly polar and hydrophobic. Figure **S6** shows the docking results, highlighting the successful reproduction of the crystallographic information *in silico*.

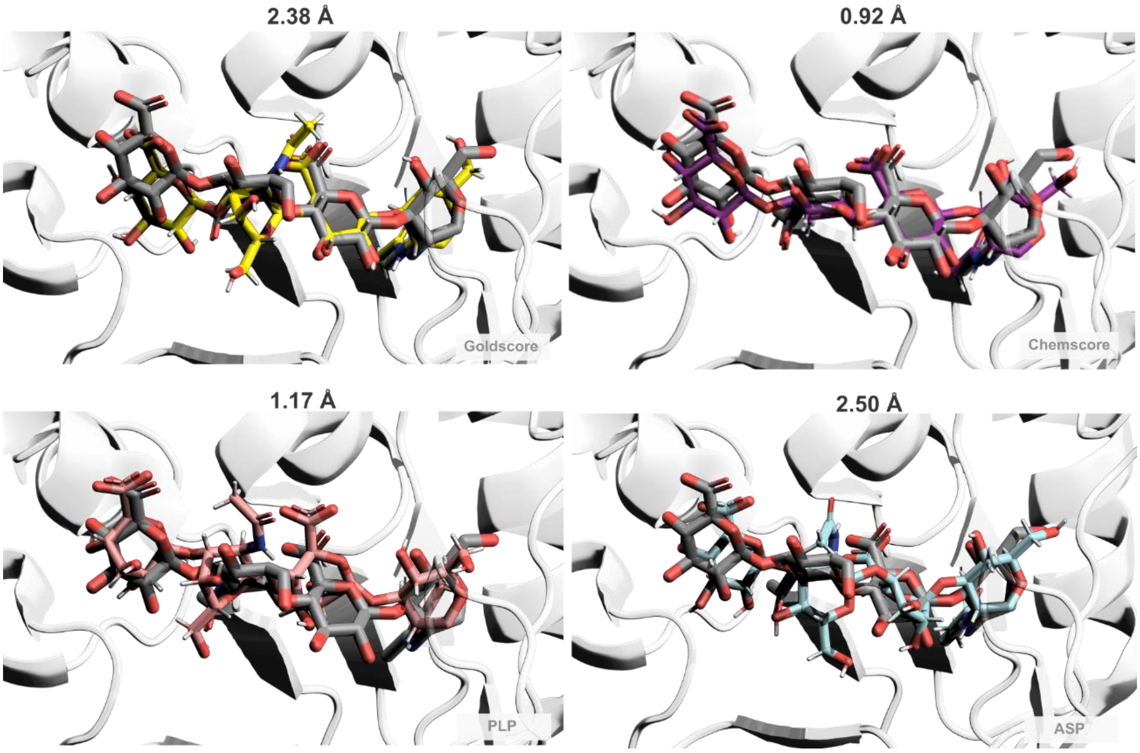

Figure S6: Pose prediction of the tetrameric product by each scoring function (GoldScore, ChemScore, ChemPLP and ASP) implemented in the GOLD software. RMSD values of the predicted poses in comparison with the crystallographic pose are presented on top of each pose.

Building upon this validated docking methodology, we proceeded to dock a pentasaccharide segment to the -4 to +1 subsites of svHyal-1-binding site. This intermediate step allowed us to identify additional interactions between the substrate and the enzyme's active site residues, particularly around the +1 subsite. We further docked another pentasaccharide segment to the -1 to +4 subsites, applying restraints based on the insights gained from the previous docking step. Finally, we docked a full octasaccharide segment, applying the same restraints, and compared the poses with the previous docking steps. The best docking pose solutions, as ranked by the ChemScore and PLP scoring functions, were selected and combined with the known binding mode of the remaining substrate to generate a complete SvHyal-1:octasaccharide complex.

Two different starting conformations for the substrate were examined, both for the Asp-COO^- and Asp-COOH models. The differences were limited to the terminal part of the substrate. As subsequent MD simulations have not resulted in significant differences, the trajectories that started from the two different conformations were concatenated.

Classical Molecular Dynamics (cMD) Simulations

Table S2: Detailed protocol for molecular dynamics simulations including energy minimization, equilibration under NVT (310.15 K) and NPT (1 bar) conditions, and an extensive production phase of 200 ns, ensuring an in-depth investigation of the enzyme-substrate dynamic behavior. Positional restraints of 1000.0 kJ.mol^{-1}.nm^{-2} were applied to the svHyal-1: hyaluronic acid (HA) complex. Harmonic potentials of 5000.0 kJ.mol^{-1}.nm^{-1} were applied to residues involved in the catalytic cycle. *t* and *b* stand for thermostat and barostat, respectively.

	**Convergence criteria** (kJ.mol^{-1}.nm^{-1})	**Frozen groups** (freezegrps)	**Positional restraints** (1000.0 kJ.mol^{-1}.nm^{-2})	**Harmonic Potentials** (5000.0 kJ.mol^{-1}.nm^{-1})	**Time**
**Minimization** Regular Lennard Jones	1000.0	SvHyal-1:HA and not hydrogens	-	-	10000 Cycles
**NVT** *(t)* V-rescale	-	-	SvHyal-1:HA	-	2 ns
**Minimization** Regular Lennard Jones	1000.0	SvHyal-1:HA backbone	-	-	30000 cycles
	500.0	-			
**NVT** *(t)* V-rescale	-	-	SvHyal-1 Backbone	**Asp-COO$^-$ model** d(C Asp110 – H$_2$N GlcA) d(C Glu112 – O4 GlcA) **Asp-COOH model** d(O Asp110 – H$_2$N GlcA) d(H Asp110 – Oε1 Glu112)	100 ps
**NPT** (t) V-rescale (b) C-rescale	-	-	SvHyal-1:HA	-	500 ps
**NPT** (t) V-rescale (b) Parrinello-Rahman	-	-	-	**Asp-COO$^-$ model** d(C Asp110 – H$_2$N GlcA) d(C Glu112 – O4 GlcA) **Asp-COOH model** d(O Asp110 – H$_2$N GlcA) d(H Asp110 – Oε1 Glu112)	50 ns
**NPT** *(t)* V-rescale *(b)* Parrinello-Rahman	-	-	-	-	200 ns

Table S3: Detailed protocol for molecular dynamics simulations using the last frame from the SMD of the glycosylation step. This protocol includes energy minimization, equilibration under NVT (310.15 K) and NPT (1 bar) conditions, and an extensive production phase of 100 ns, ensuring an in-depth investigation of the enzyme-substrate dynamic behavior. Positional restraints of 1000.0 $kJ.mol^{-1}.nm^{-2}$ were applied to the SvHyal-1:hyaluronic acid (HA) complex. Harmonic potentials of 5000.0 $kJ.mol^{-1}.nm^{-1}$ were applied to residues involved in the catalytic cycle. *t* and *b* stand for thermostat and barostat, respectively.

<table>
<tr><th></th><th>Convergence criteria ($kJ.mol^{-1}.nm^{-1}$)</th><th>Frozen groups (freezegrps)</th><th>Positional restraints (1000.0 $kJ.mol^{-1}.nm^{-2}$)</th><th>Harmonic Potentials (2000.0 $kJ.mol^{-1}.nm^{-1}$)</th><th>Time</th></tr>
<tr><td>Minimization
Regular Lennard Jones</td><td>1000.0</td><td>SvHyal-1:OXA and not hydrogens</td><td>-</td><td>-</td><td>10000 Cycles</td></tr>
<tr><td>NVT
(t) V-rescale</td><td>-</td><td>-</td><td>SvHyal-1:HA</td><td>-</td><td>2 ns</td></tr>
<tr><td rowspan="2">Minimization
Regular Lennard Jones</td><td>1000.0</td><td>SvHyal-1:OXA backbone</td><td rowspan="2">-</td><td rowspan="2">-</td><td rowspan="2">30000 cycles</td></tr>
<tr><td>500.0</td><td>-</td></tr>
<tr><td>NVT
(t) V-rescale</td><td>-</td><td>-</td><td>SvHyal-1 Backbone</td><td>Asp-COOH model
d(H Asp110 – N OXA_{427})
d(C Glu112 – C_{anom} OXA_{427})</td><td>100 ps</td></tr>
<tr><td>NPT
(t) V-rescale
(b) C-rescale</td><td>-</td><td>-</td><td>SvHyal-1:OXA</td><td>-</td><td>500 ps</td></tr>
<tr><td>NPT
(t) V-rescale
(b) Parrinello-Rahman</td><td>-</td><td>-</td><td>-</td><td>Asp-COOH model
d(H Asp110 – N OXA_{427})
d(C Glu112 – C_{anom} OXA_{427})</td><td>50 ns</td></tr>
<tr><td>NPT
(t) V-rescale
(b) Parrinello-Rahman</td><td>-</td><td>-</td><td>-</td><td>-</td><td>100 ns</td></tr>
</table>

In summary, we enclosed the system in a box measuring 105.25 × 105.25 × 105.25 $Å^3$ filled with TIP3P water molecules and relaxed it in several stages. First, we relaxed all water molecules and hydrogen atoms using standard Lennard-Jones minimization. This was followed by a 2 ns equilibration phase at 310.15 K, during which the SvHyal-1 enzyme and HA substrate were constrained. Next, the entire system underwent minimization and was then equilibrated for 100 ps with SvHyal-1 backbone constrained. During this equilibration, the system was heated to reach the target temperature of 310.15 K. The simulation continued in the NPT ensemble for an additional 500 ps with the SvHyal-1:HA constrained, followed by 50 ps with harmonic potentials applied to the catalytic residues (Asp110 and Glu112 with the GlcNAc unit at subsite -1). Finally, we conducted a 200 ns production run across 4 replicas.

QM/MM MD Umbrella Sampling simulations

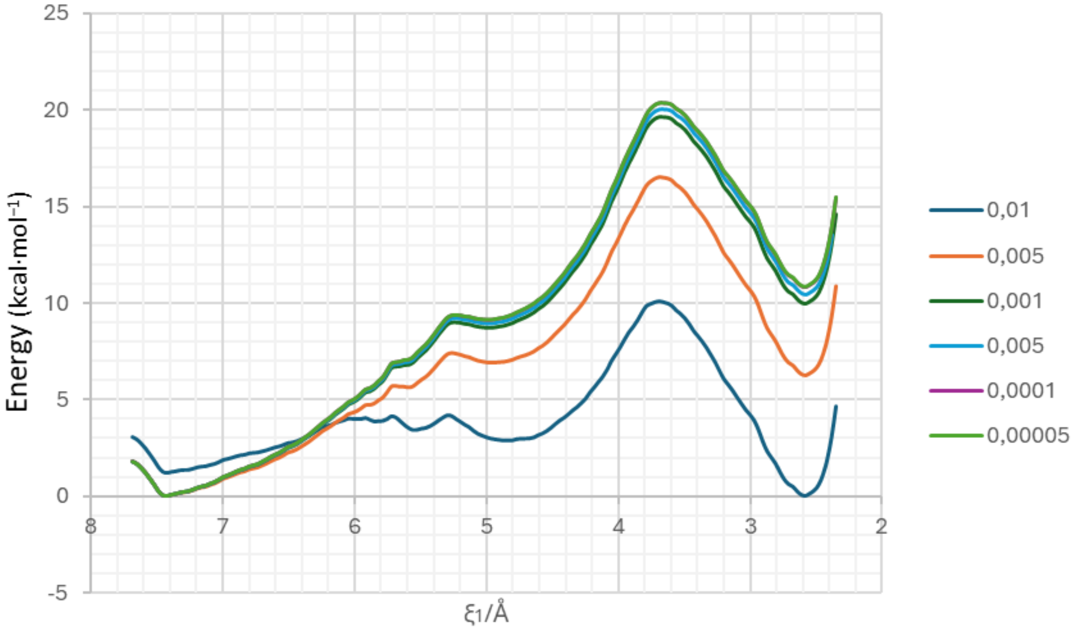

Figure S7: Free energy profiles calculated using WHAM along the first reaction coordinate (RC_1), with different convergence tolerance parameter values. The chosen convergence tolerance value was 0.0001.

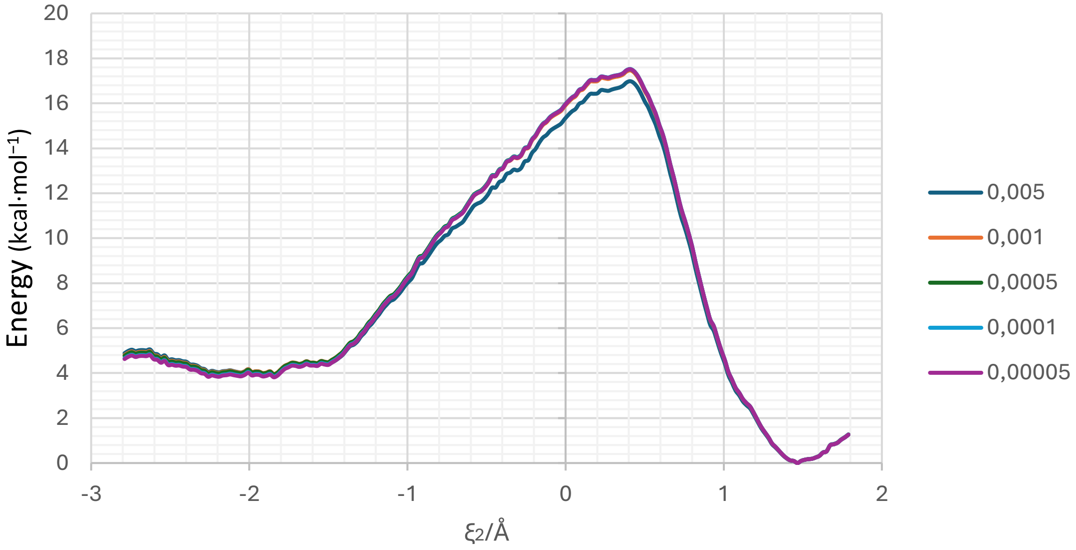

Figure S8: Free energy profiles calculated using WHAM along the second reaction coordinate (RC_2), with different convergence tolerance parameter values. The chosen convergence tolerance value was 0.001.

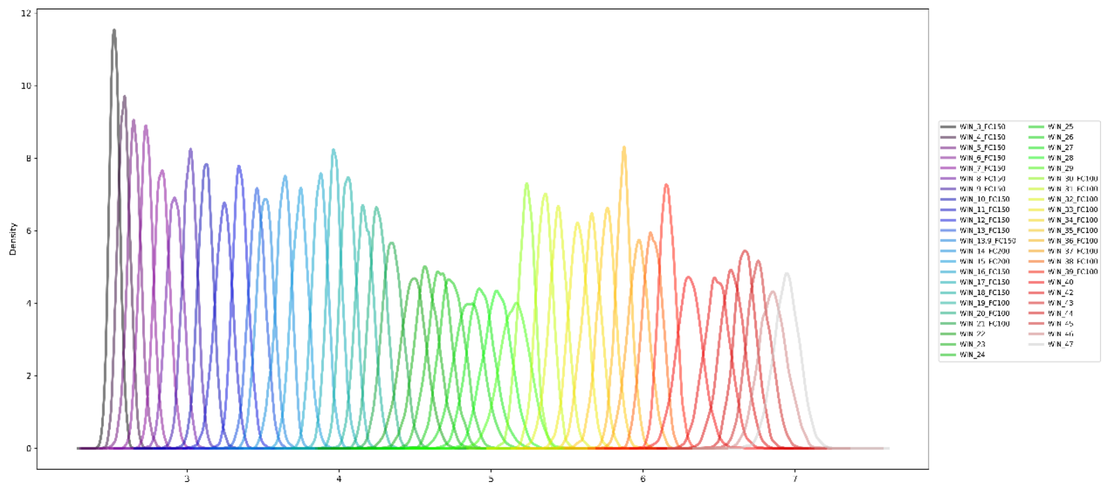

Figure S9: Density distributions of configuration samples within the US windows for the RC_1 (glycosylation step) of the Asp-COO^- model.

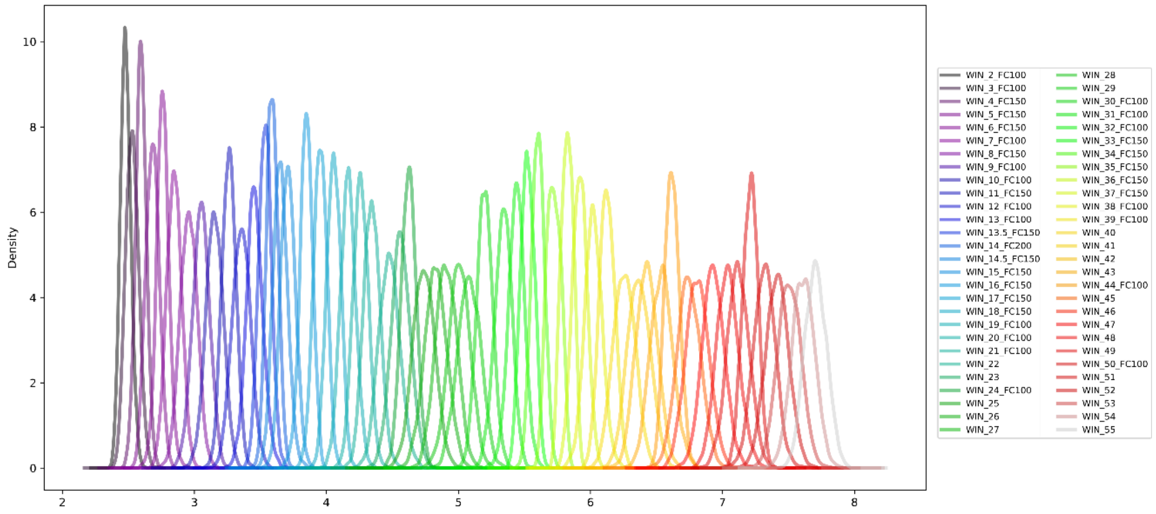

Figure S10: Density distributions of configuration samples within the US windows for RC_1 (glycosylation step) of the Asp-COOH model.

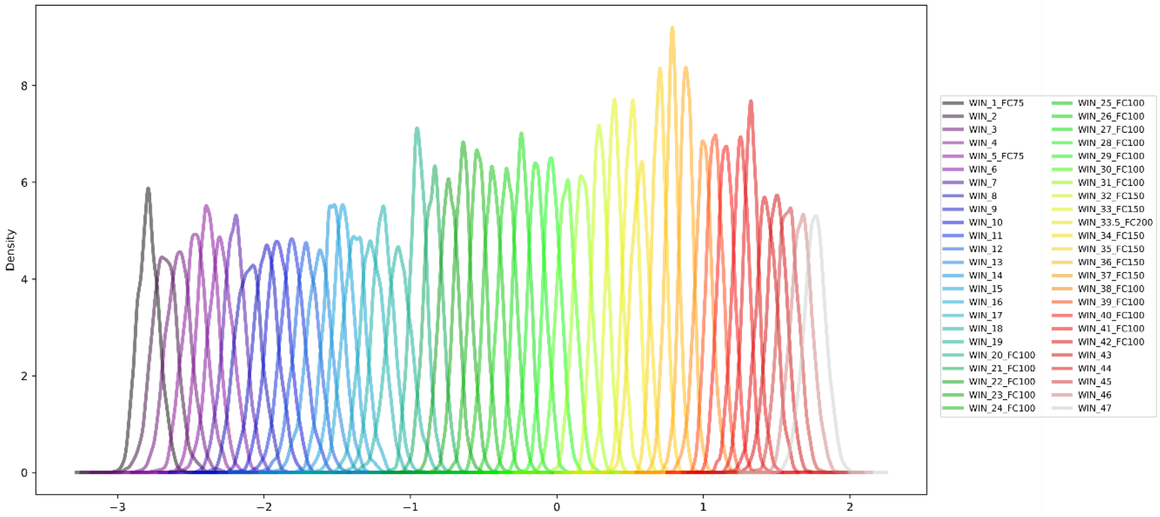

Figure S11: Density distributions of configuration samples within the US windows for RC_2 (deglycosylation step) of the Asp-COOH model.

a)

b)

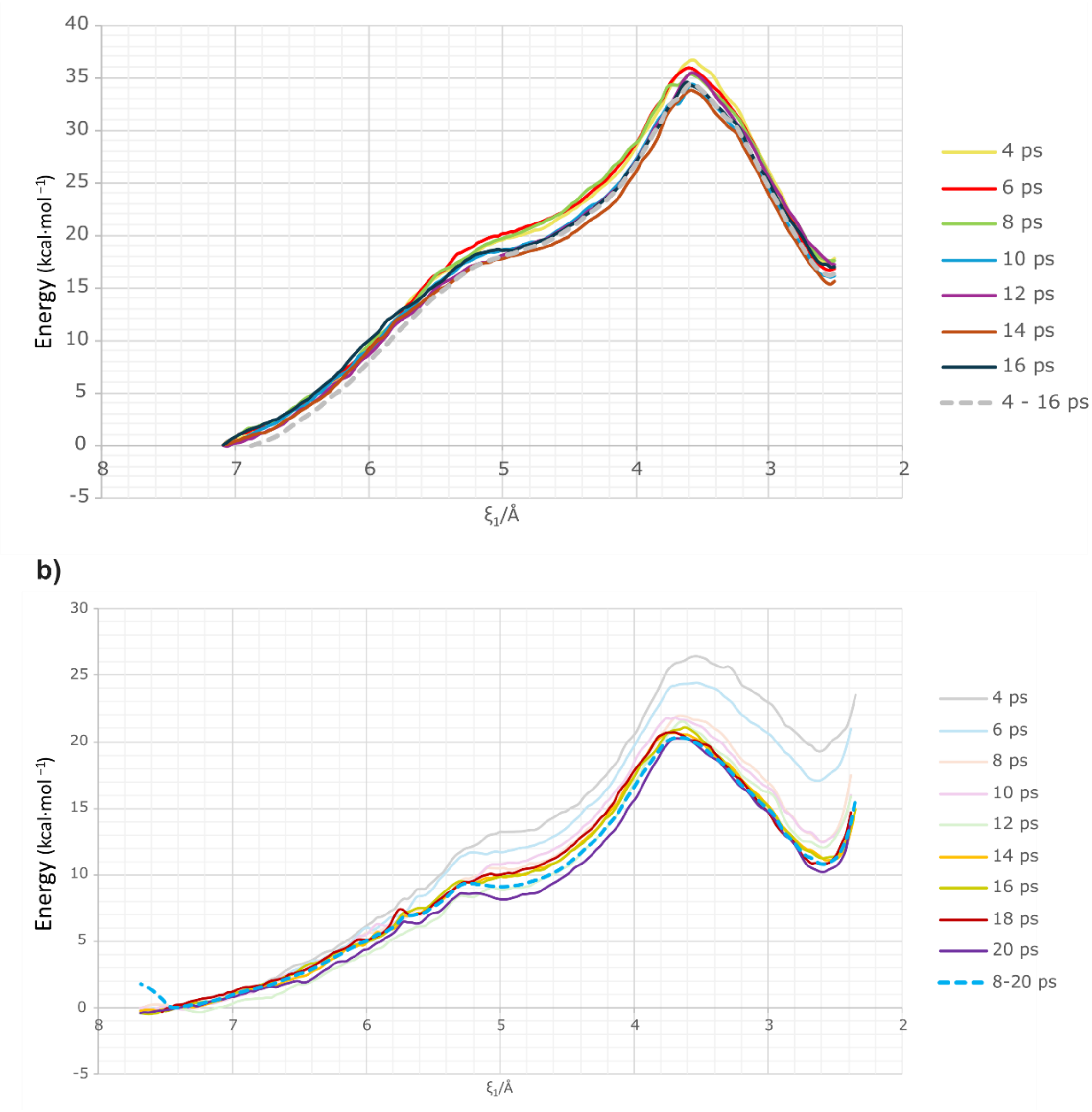

Figure S12: Evolution of PMFs over blocks of 2 ps, along the RC_1 (glycosylation step) to assess convergence, for a) Asp-COO- and b) Asp-COOH models.

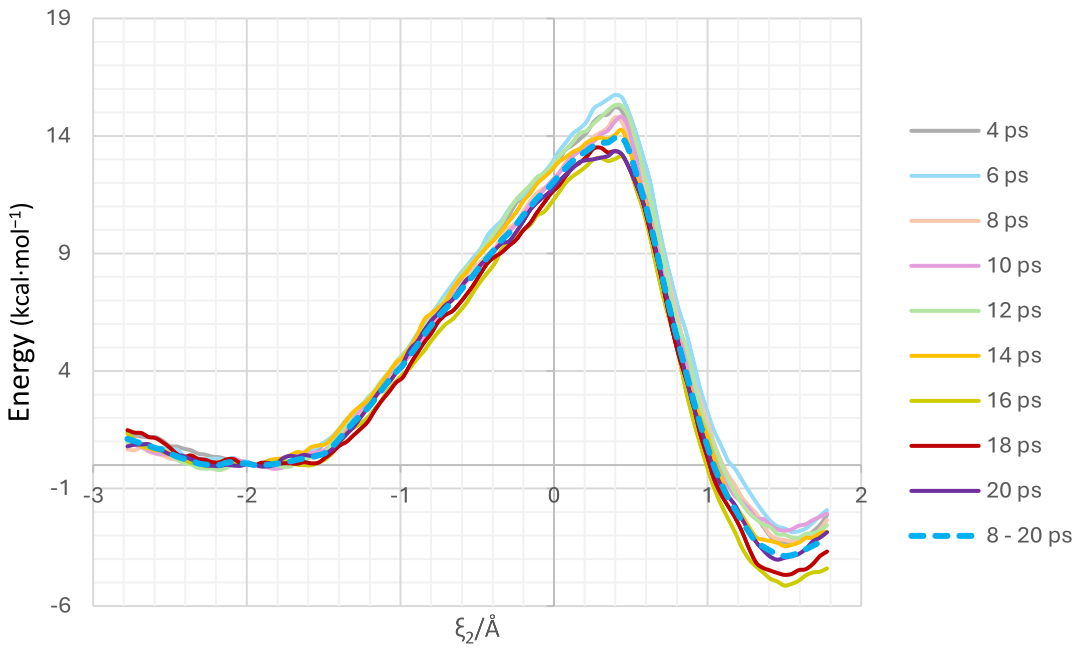

Figure S13: Evolution of PMFs over blocks of 2 ps, along RC_2 (deglycosylation step) of the Asp-COOH model to assess convergence.

Section III – Results and Discussion

Assessing the dynamic behaviour of Asp-COO$^-$ and Asp-COOH models

Notably, in both models, the enzyme exhibited overall stability throughout the last 200 ns of the simulation trajectory compared to the initial structure. The average root-mean-square deviation (RMSD) values ranged from 1.54 Å to 1.45 Å, for Asp-COO$^-$ and Asp-COOH models, respectively (**Figure S14A and Figure S15A**). As expected, the octasaccharide substrate underwent significant structural rearrangements as it adapted to the enzyme's active site cleft and interacted with the surrounding solvent. **Figure S14B and Figure S15B** show the different conformational positions that the substrate adopted along the trajectory, depicted from 200 to 200 frames. Moreover, when analyzing the RMSD of the octasaccharide, one may infer that the region that corresponds to the bvHyal tetrasaccharide product (subsites -4 to -1, non-reducing end) underwent fewer fluctuations/rearrangements than the modeled portion/leaving group (subsites +1 to +4, reducing end) (**Figure S14B and S15B**).

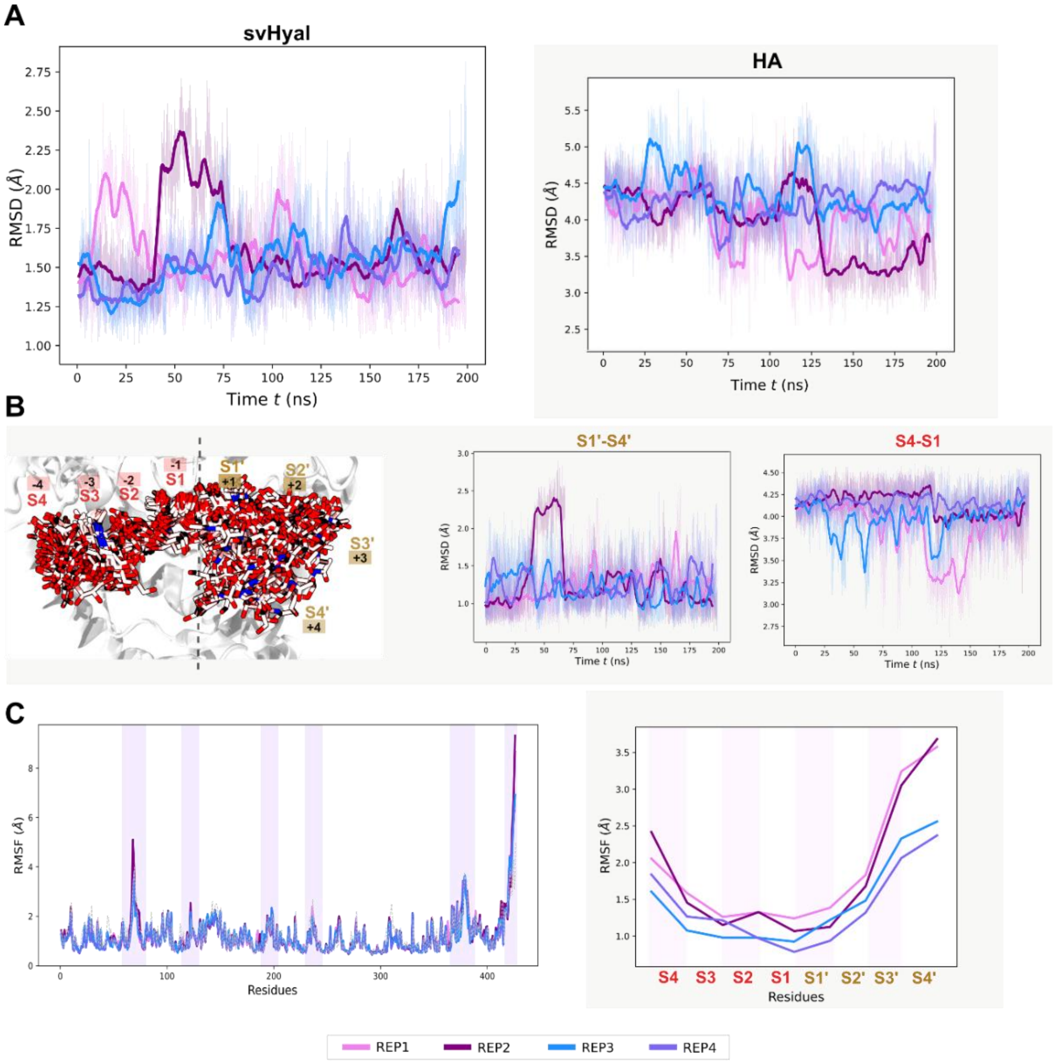

Figure S14: Qualitative and quantitative analysis of the MD simulations across four replicas of the Asp-COO$^-$ model with respect to the initial structure. (A) RMSD analysis of the SvHyal-1 and hyaluronic acid backbone. (B) Depiction of substrate configurations sampled every 200 frames throughout the trajectory,

along with RMSD values for the known non-reducing end (-4 to -1) and the unknown reducing ends (+1 to +4). (C) RMSF of SvHyal-1 (left) for each replica and the apo structure (dashed grey). The purple shadow denotes the regions that underwent significant fluctuations (residues 63 to 75, 118 to 126, 191 to 200, 231 to 240, 366 to 383, and the C-terminal end). RMSF values for the substrate octasaccharide are also shown (right). Color scheme: REP1 (pink), REP2 (magenta), REP3 (blue), and REP4 (violet).

The analysis of the root-mean-square fluctuation (RMSF) indicated that the overall fluctuations of SvHyal-1 were slightly less pronounced when compared to the apo structure. However, these fluctuations didn’t reflect significant changes in the enzyme's structure upon hyaluronic acid-substrate binding, except for a slight closure of the active site pocket due to the movement of a loop (residues 64 to 73), highlighted in the RMSF results. This analysis suggests that the substrate, although to a small extent, conferred stabilization to the enzyme (**Figure S14C and Figure S15C**).

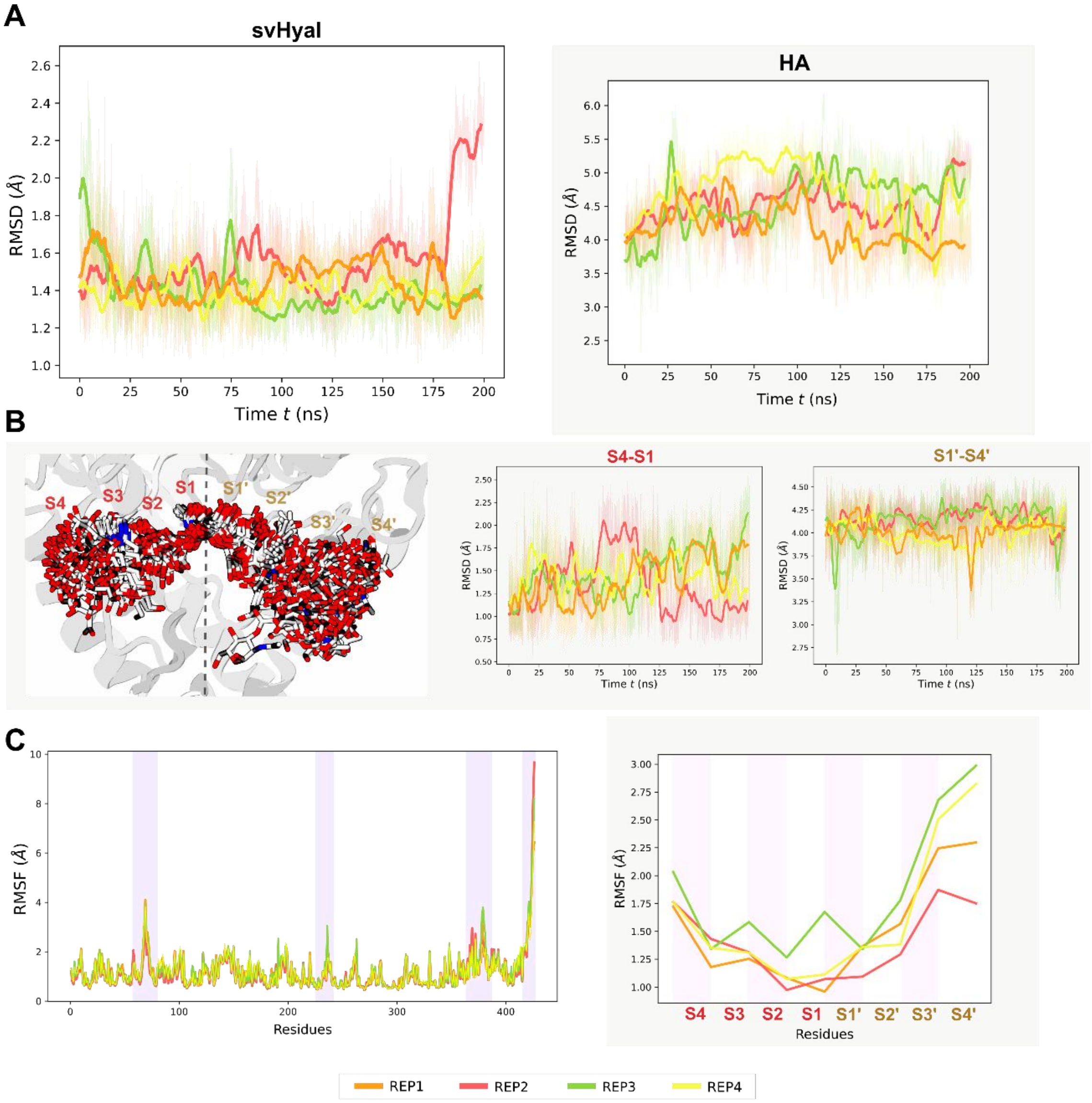

Figure S15: Qualitative and quantitative analysis of the MD simulations across four replicas of the Asp-COOH model concerning the initial structure. (A) RMSD analysis of the SvHyal-1 and hyaluronic backbone. (B) Depiction of substrate configurations sampled every 200 frames throughout the trajectory, along with

RMSD values for the known non-reducing end (-4 to -1) and the unknown reducing ends (+1 to +4). (C) RMSF of SvHyal-1 (left) for each replica. The purple shadow denotes the regions that underwent significant fluctuations (residues 63 to 75, 231 to 240, 366 to 383, and the C-terminal end). RMSF values for the substrate octasaccharide are also shown (right). Color scheme: REP1 (pink), REP2 (magenta), REP3 (blue), and REP4 (violet).

Additionally, corroborating the RMSD results, the RMSF for the substrate exhibited higher fluctuations on the reducing end (+1 to +4) than for the non-reducing end (-4 to -1). One may infer that the reducing end is much more unstable than the non-reducing one, which might be due to weaker interactions as well as interactions with the solvent.

The distances between the catalytic (Glu112) and stabilizing residues (Asp110, Tyr183, Tyr230) with the hyaluronic acid octasaccharide substrate were also calculated throughout the trajectory. Regarding the Asp-COO^- model (**Figure 16**), we observed that the distance between the catalytic glutamic acid residue and the glycosidic oxygen (Glu112 Oε2-O_{glyc}) was overall stable in REP2 and REP3, with a mean distance of 4.00 Å and 3.89 Å, respectively. In these two replicas, the measured distances have remained stable and suitable for the mechanistic study.

Additionally, throughout the trajectory, the Glu112 revealed a higher preference for the *anti-* over the *syn*-configuration, despite the latter being thought to be energetically more favorable for the reaction. However, *Pengthaisong et. al.* [2], reported that retaining GH structures follow either a syn- or *anti*-protonation mechanism, based on the position of the active site catalytic acid/base residue with respect to the endocyclic oxygen within the sugar ring. In this specific case, the preference for an *anti*-conformation arises from the interaction of the anti-Glu_{112} with its neighboring stabilizing residue, Asp110.

Additionally, the oxygen in the 2-acetamido group ($O2N_{NAc}$), acting as the catalytic nucleophile, was found to be at an average distance of 3.0 Å from the anomeric C1 carbon ($O2N_{Nac}$-C_{anom}). This positioning is critical in the substrate binding process for effective hydrolysis.

Therefore, based on the calculated distances and the observed higher stability of both the SvHyal-1 and the hyaluronic acid substrate in the RMSD plots, we selected REP2 for further studies regarding the Asp-COO^- model.

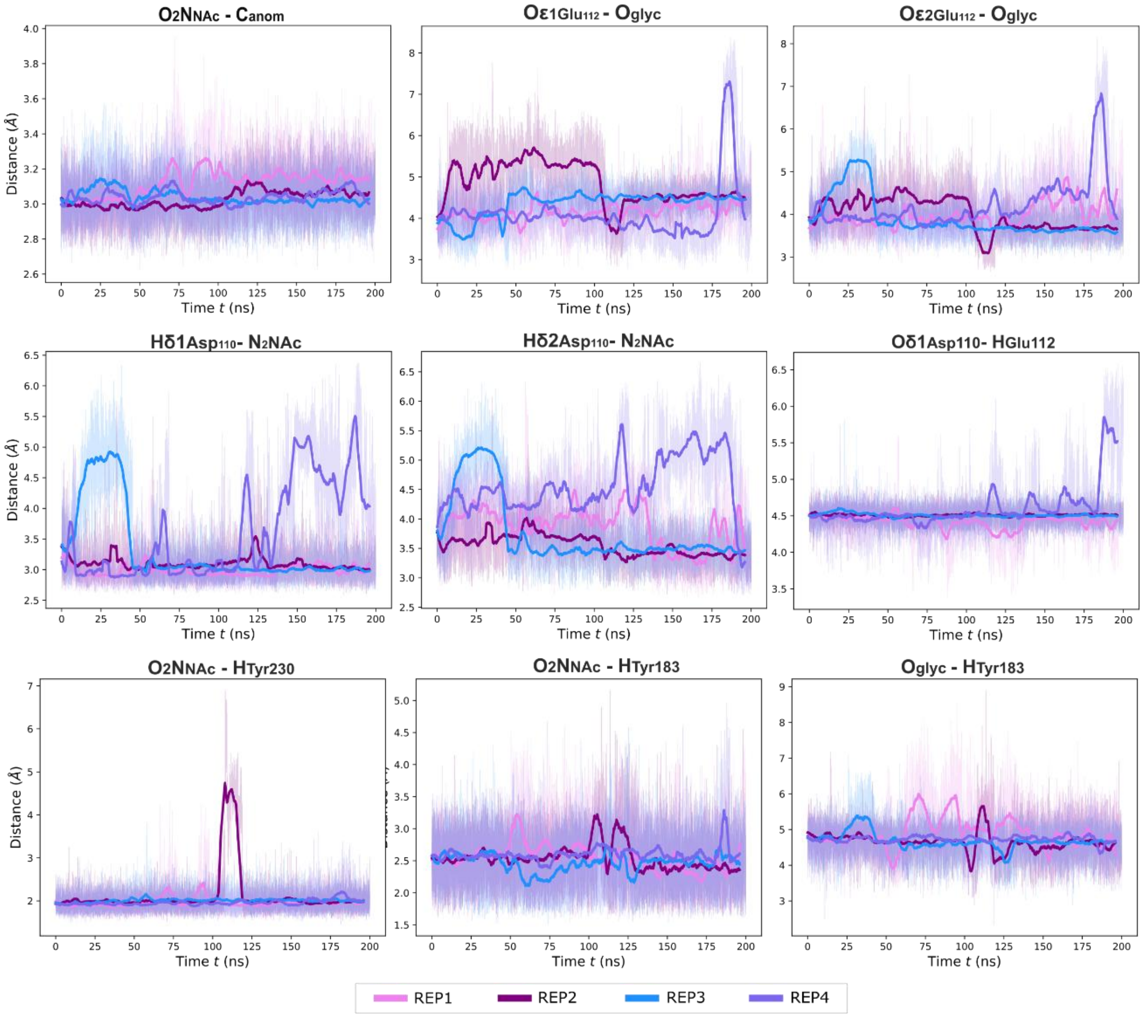

Figure S16: Distance variation throughout the last 200 ns MD simulation across the four replicas of the Asp-COO^- model. The solid lines were calculated using the *gmx mindist* tool from GROMACS package.

Considering the Glu112 Oε2 -O_{glyc} in the Asp-COOH model (**Figure S17**), it is noteworthy that REP1 and REP4 exhibited higher stability, characterized by a mean distance of 3.54 Å and 3.35 Å, respectively. Nevertheless, although the other distances remained relatively constant throughout the simulation, the distance between Asp_{110} and the N-acetyl group of the hyaluronic acid substrate only achieved an appropriate distance for interaction in REP1. One of the oxygen atoms of the Asp110 carboxylate group is expected to interact with the nitrogen in the N-acetyl group, as this interaction is believed to stabilize and position the substrate in a catalytic orientation and for intermediate stabilization. This observation, combined with the noted stability in the previous analysis, prompted us to select this specific replica, REP1, for subsequent analyses for the Asp-COOH model.

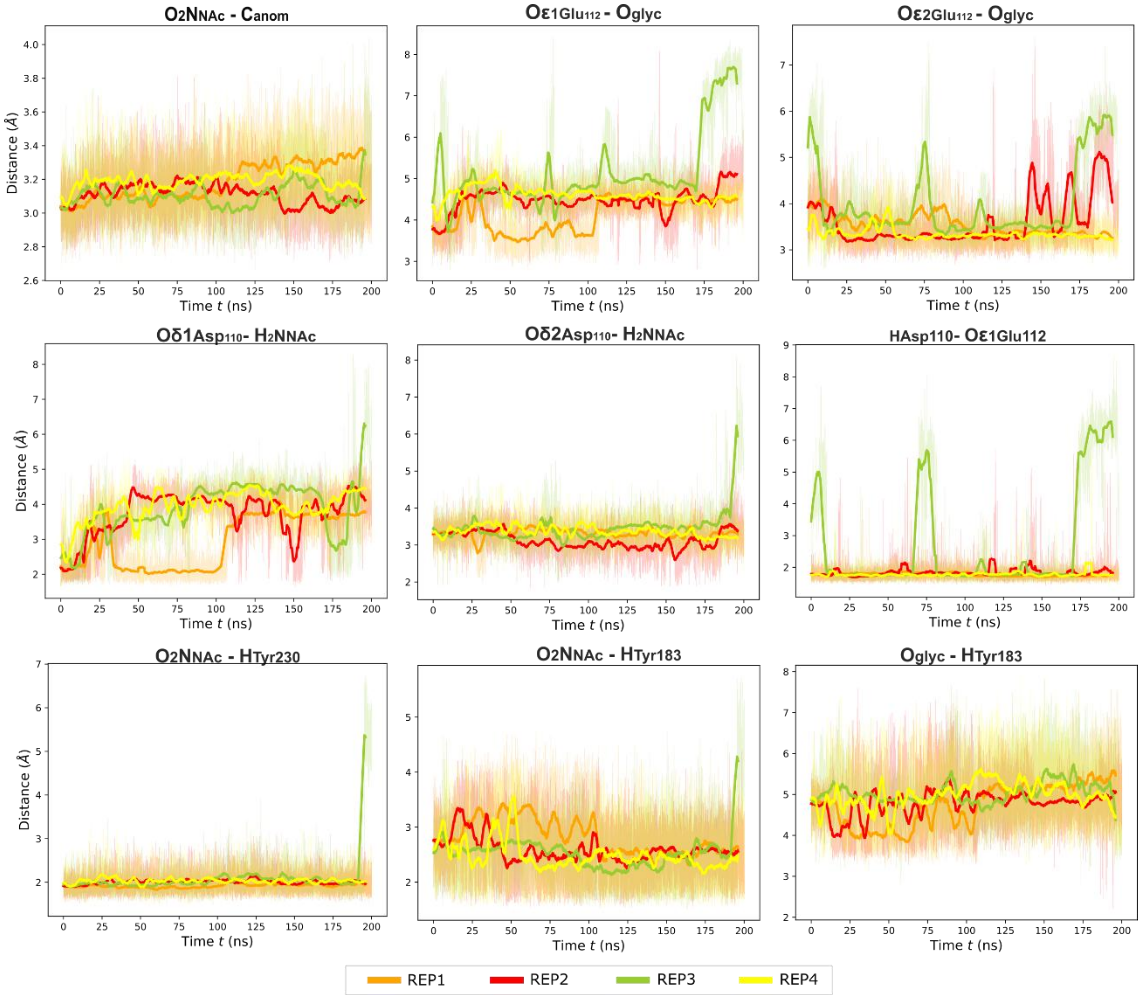

Figure S17: Distance variation throughout the last 200 ns MD simulation across the four replicas of the Asp-COOH model. The solid lines were calculated using the *gmx mindist* tool from GROMACS package.

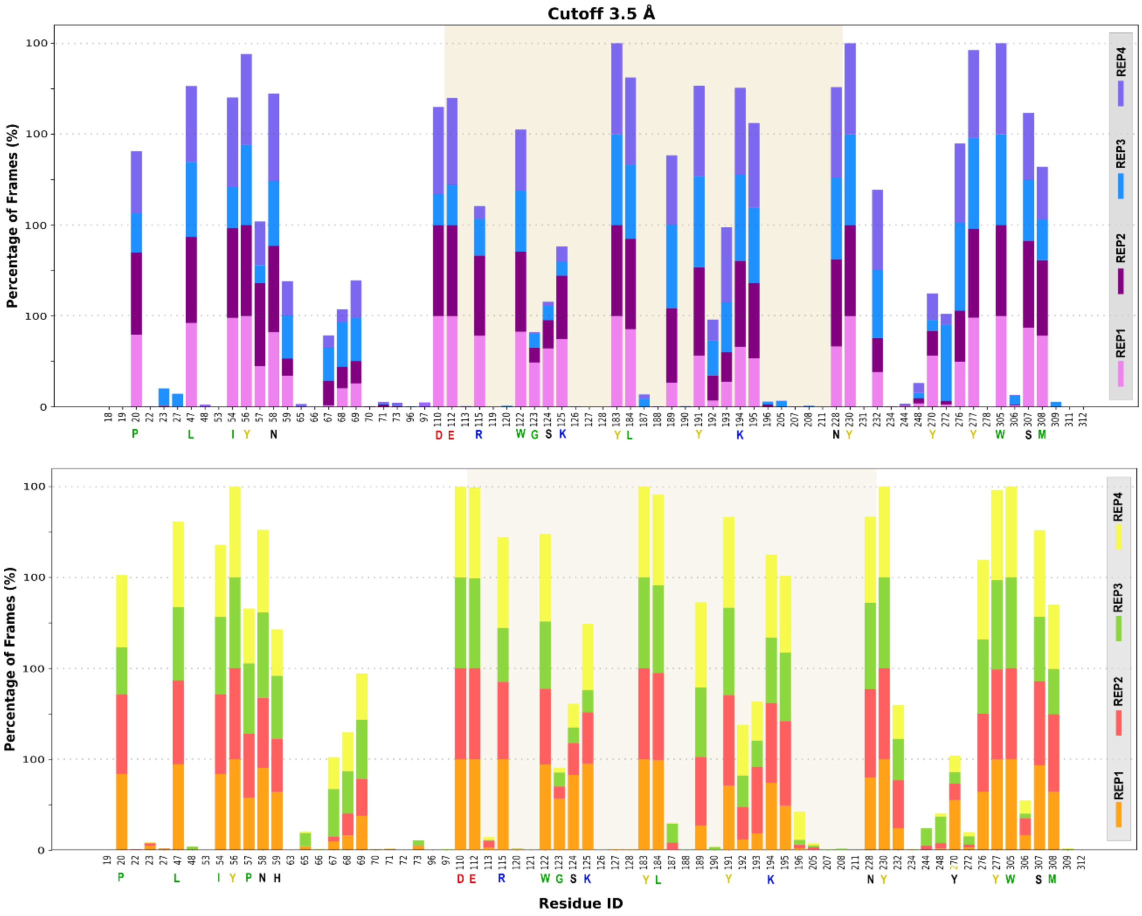

Figure S18: Percentage of contacts with a 3.5 Å cutoff between SvHyal-1 residues and the hyaluronic acid substrate across the four replicas for the Asp-COO^- (top) and Asp-COOH (bottom) models. Only the residues that established contacts are plotted. Residues that maintained contact over 50% of the trajectory are identified (negatively charged – red; positively charged – blue; hydrophilic – black; tyrosines – yellow and hydrophobic – green). Residues that interact with the reducing end (leaving group) of the substrate are shaded in faded yellow.

Table S4: Values of the Cremer–Pople ring-puckering angles (φ, θ, Q) for the HA GlcNAc at subunit -1, before docking (unbound HA), after docking (SvHyal-1:hyaluronic acid), and during MD. Values were obtained from the concatenated trajectories of Asp-COO^- and Asp-COOH models. All angles are expressed in degrees (°).

Unbound hyaluronic acid	SvHyal-1:hyaluronic acid	During MD
φ = 230.86	φ = 240.58	φ = 230.94 ± 9.18
θ = 1.22	θ = 89.68	θ = 83.48 ± 5.13
Q = 0.56	Q = 0.77	Q = 0.65 ± 0.05
$^4C_1$	$^{1,4}B/^1S_3$	$^{1,4}B/^1S_3$

Reaction Mechanism – Step 1: Glycosylation

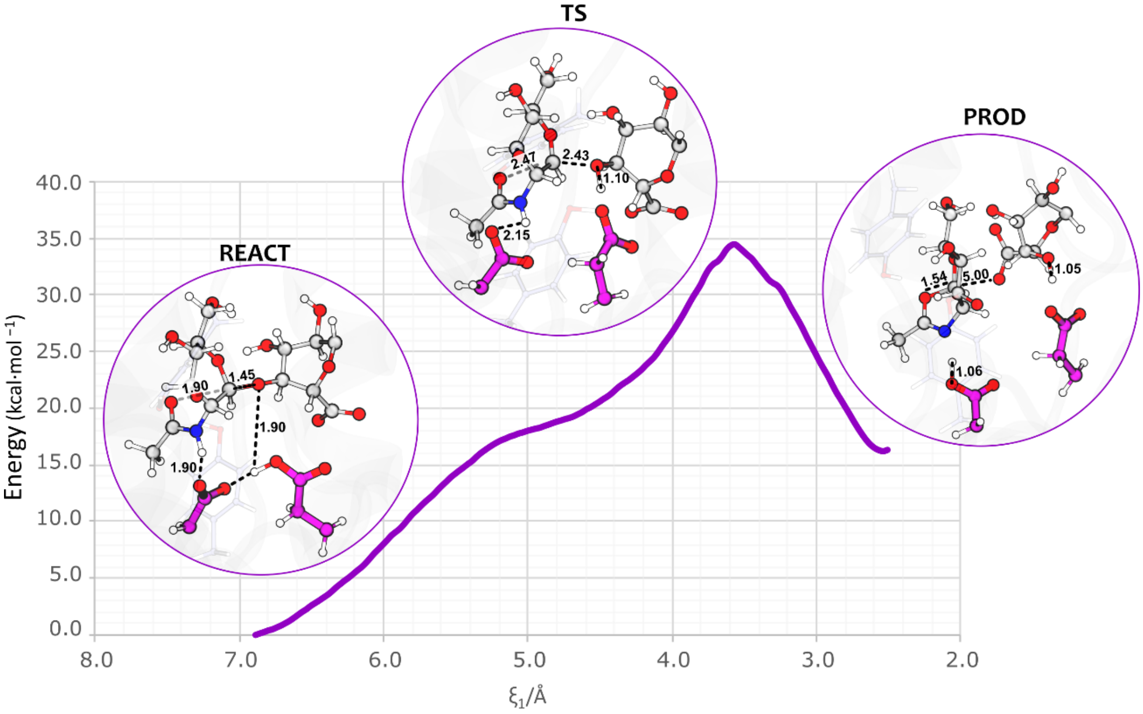

Figure S19: Stick representation of the representative structures corresponding to different stationary points along the glycosylation step in the ASP-COO- model. A bold purple line represents the corresponding PMF.

Table S5*:* Changes in average bond distances during the glycosylation reaction pathway for the Asp-COO^- (pink) and the Asp-COOH (orange) models, and respective standard deviations.

	$O4_{glyc}$-$H^{\varepsilon 2}_{Glu112}$	C^{1}_{anom}-O^{2N}_{Nac}	C^{1}_{anom}-O_{glyc}	H^{2N}_{NAc}-$O^{\delta 1}_{Asp110}$	H^{2N}_{NAc}-N^{2}_{NAc}	O^{2N}_{Nac}-H_{Tyr230}
**COO^-**						
**REACT**	3.98 ± 0.15	2.87 ± 0.11	1.45 ± 0.04	1.90 ± 0.27	1.05 ± 0.04	1.72 ± 0.16
**TS**	1.10 ± 0.09	2.47 ± 0.08	2.43 ± 0.67	2.15 ± 0.57	1.09 ± 0.06	1.84 ± 0.17
**INT**	1.05 ± 0.04	1.54 ± 0.06	5.00 ± 0.42	1.06 ± 0.06	1.60 ± 0.14	2.10 ± 0.29
**COOH**						
**RS**	4.16 ± 0.17	3.00 ± 0,14	1.42 ± 0.04	2.14 ± 0.23	1.04 ± 0.03	1.72 ± 0.22
**TS_RS**	2.28 ± 0.16	2.94 ± 0.19	1.43 ± 0.04	2.02 ± 0.26	1.04 ± 0.02	1.72 ± 0.15
**REACT**	2.00 ± 0.20	3.02 ± 0.14	1.42 ± 0.04	2.15 ± 0.28	1.04 ± 0.02	1.74 ± 0.16
**TS1**	1.05 ± 0.05	2.60 ± 0.07	2.24 ± 0.35	1.95 ± 0.19	1.05 ± 0.04	1.80 ± 0.16
**INT1**	1.00 ± 0.03	1.60 ± 0.07	3.22 ± 0.29	1.56 ± 0.30	1.20 ± 0.25	2.12 ± 0.24

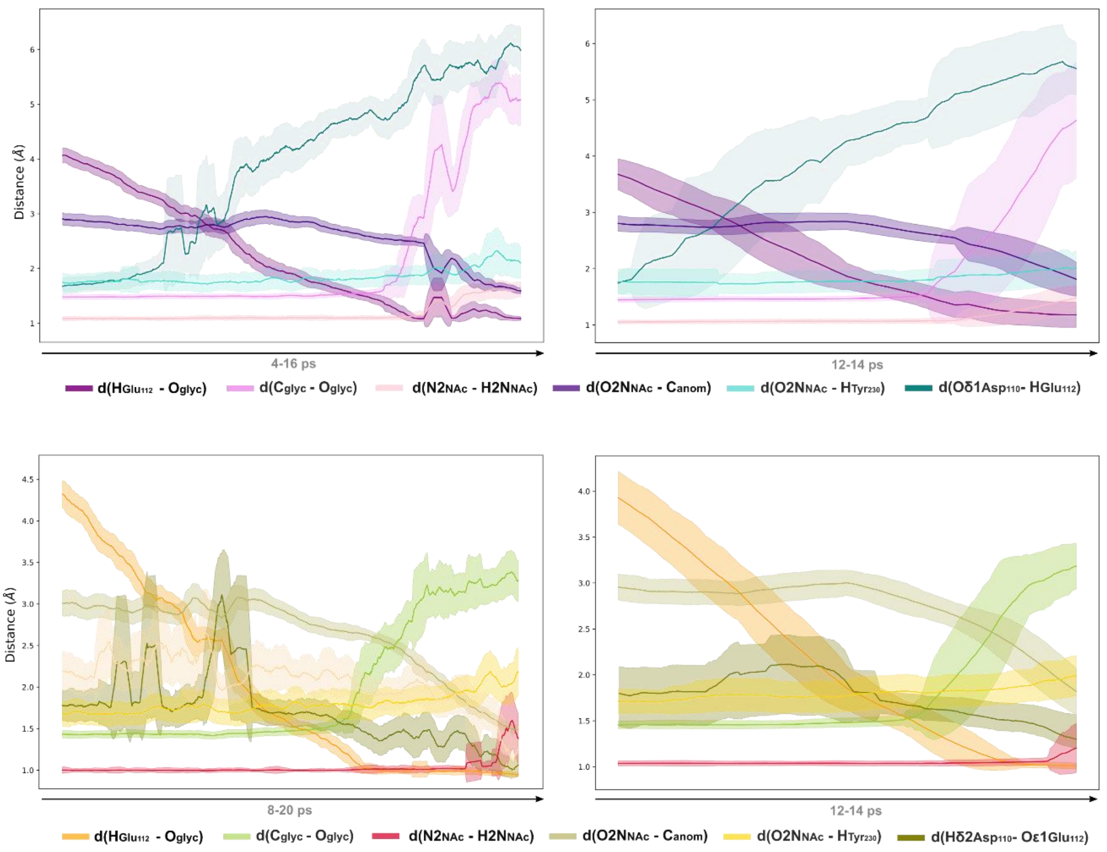

Figure S20: Time evolution of the atomic distances of key catalytic residues along the ξ1 reaction coordinate for the Asp-COO$^-$ model **(top)** and the Asp-COOH model (**bottom**); On the left, the distances along the entire trajectory after equilibration are depicted, while on the right, only a block of 12 to 14 ps is shown for better visualization.

Table S6: Changes in average sugar-ring distances during the glycosylation reaction pathway for the Asp-COOH model with standard deviations.

**Å**	**RS**	**TS_RS**	**REACT**	**TS1**	**INT1**
**C1$_{anom}$-O$_{glyc}$**	1.42 ± 0.04	1.43 ± 0.04	1.42 ± 0.04	2.24 ± 0.35	3.22 ± 0.29
**C1$_{anom}$-O5**	1.45 ± 0.04	1.46 ± 0.04	1.47 ± 0.05	1.31± 0.04	1.35 ± 0.03
**C1$_{anom}$-O2N**	3.00 ± 0,14	2.94 ± 0.19	3.02 ± 0.14	2.60 ± 0.07	1.60 ± 0.07
**C2N-O2N**	1.26 ± 0.02	1.27 ± 0.02	1.26 ± 0.02	1.27 ± 0.03	1.34 ± 0.03
**C2N-N2**	1.37 ± 0.03	1.37 ± 0.03	1.37 ± 0.03	1.37 ± 0.03	1.32 ± 0.02

Table S7: Hirshfield charges distribution along the reaction coordinate for the intervening atoms in the glycosylation step of the Asp-COOH model.

	RS	TS1	INT1
**Glu112 H**	0.600	0.597	0.572
**$C1_{anom}$**	0.053	0.039	0.063
**N2 NAc**	-0.593	-0.55	-0.474
**Asp Oδ2**	-0.577	-0.640	-0.704
**Glu Oδ2**	-0.575	-0.739	-0.749
**O_{glyc}**	-0.574	-0.781	-0.851
**$O5_{ring}$**	-0.543	-0.369	-0.474
**O2N NAc**	-0.648	-0.620	-0.486

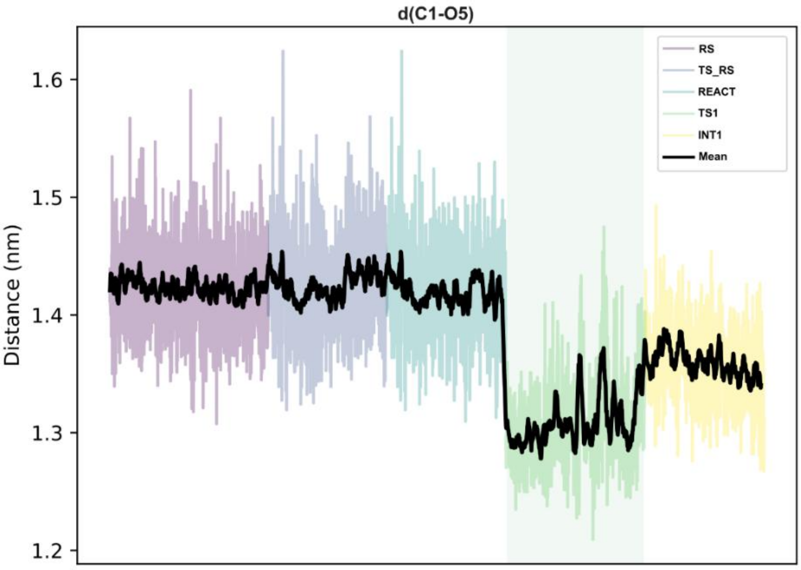

Figure S21: Evolution of the GlcNAc C1-O5 distance along the glycosylation reaction in the Asp-COOH model.

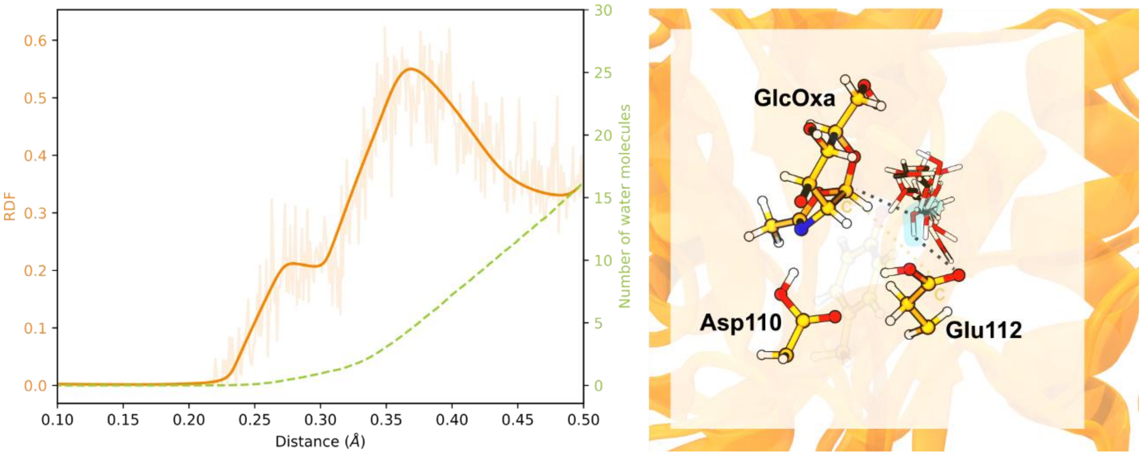

Figure S22: Radial distribution function of water oxygen atoms around the midpoint between C Oxa – C Glu112 (left) and three-dimensional representation of the solvation sphere around GlcOxa and Glu112 (right).

The radial distribution function (RDF) calculated between the midpoint of the oxazoline carbon and the carboxyl carbon of Glu112 revealed a first solvation shell centered around 3.0 Å, corresponding on average to roughly 2 water molecules. A second, broader solvation shell was observed at around 4.75 Å, consistent with the bulk solvent.

Reaction Mechanism – Step 2: Deglycosylation

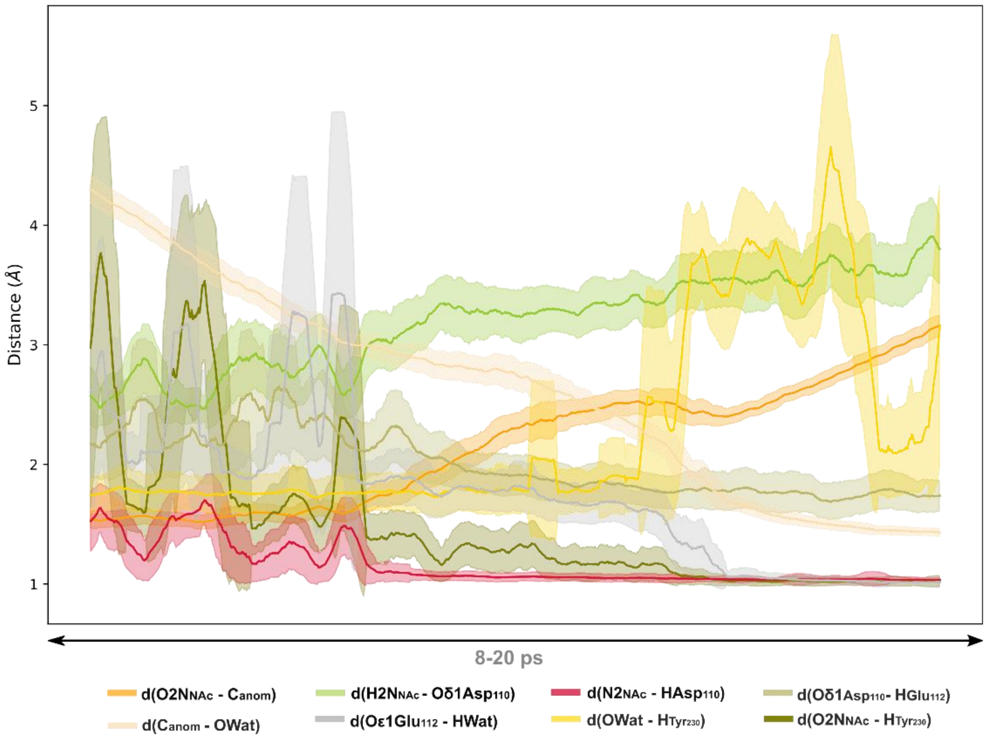

Figure S23: Time evolution of the atomic distances of key catalytic residues along the ξ2 reaction coordinate for the Asp-COOH deglycosylation step; Distances along the entire trajectory after equilibration are depicted.

Table S8: Changes in average bond distances during the deglycosylation reaction pathway for the Asp-COOH model, and respective standard deviations in Å. *IS stands for initial structure before the minimum in the PMF.

Distances (Å)	IS	INT2	TS2	PROD
**$C1_{anom}$ – O2N Oxa**	1.56 ± 0.07	1.58 ± 0.07	2.50 ± 0.12	2.95 ± 0.09
**Oδ1 Asp110 – H Glu112**	1.74 ± 0.46	1.61 ± 0.51	1.12 ± 0.12	1.02 ± 0.04
**H Asp110 – N2 Oxa**	1.33 ± 0.23	1.25 ± 0.22	1.05 ± 0.04	1.04 ± 0.04
**H Tyr230 – O2N Oxa**	2.41 ± 0.46	2.40 ± 0.47	1.78 ± 0.14	1.77 ± 0.15
**H Tyr183 – O Wat**	1.78 ± 0.16	1.77 ± 0.17	2.73 ± 0.99	2.31 ± 0.71
**Owat – $C1_{anom}$**	4.04 ± 0.13	3.48 ± 0.12	2.11 ± 0.18	1.45 ± 0.04
**H Wat – Oε1 Glu112**	2.06 ± 0.37	1.94 ± 0.27	1.48 ± 0.24	1.02 ± 0.04

Table S9: Values of the Cremer–Pople ring-puckering angles (φ, θ, Q) for the hyaluronic acid GlcNAc at subunit -1 across the glycosylation (top) and deglycosylation (bottom) steps. The ASP-COO^- and ASP-COOH models are represented by pink and orange colors, respectively. All angles are expressed in degrees (º).

Asp-COO^-

REACT	TS	PROD
φ = 249.49 ± 21.20	φ = 244.09 ± 18.47	φ = 265.74 ± 24.83
θ = 67.68 ± 10.48	θ = 48.80 ± 7.14	θ = 27.46 ± 7.68
Q = 0.59 ± 0.05	Q = 0.48 ± 0.06	Q = 0.51 ± 0.05

Asp-COOH

RS	TS_RS	REACT	TS1	INT1
φ = 238.67 ± 15.10	φ = 250.83 ± 17.60	φ = 245.44 ± 23.23	φ = 243.99 ± 12.97	φ =240.64 ± 21.31
θ = 74.99 ± 9.11	θ = 70.28 ± 12.00	θ = 68.84 ± 14.56	θ = 56.51 ± 8.12	θ = 27.06 ± 6.40
Q = 0.64 ± 0,06	Q = 0.62 ± 0.06	Q = 0.61 ± 0.06	Q = 0.53 ± 0.06	Q = 0.52 ± 0.04

IS	INT2	TS2	PROD
φ = 250.34 ± 29.80	φ = 239.31 ± 26.00	φ = 215.44 ± 13.24	φ =302.13 ± 52.22
θ = 28.61 ± 10.19	θ = 32.58 ± 13.38	θ = 57.94 ± 8.93	θ = 22.40 ± 6.48
Q = 0.53 ± 0.06	Q = 0.54 ± 0.07	Q = 0.54 ± 0.06	Q = 0.60 ± 0.05

References

(1) Marković-Housley, Z.; Miglierini, G.; Soldatova, L.; Rizkallah, P. J.; Müller, U.; Schirmer, T. Crystal Structure of Hyaluronidase, a Major Allergen of Bee Venom. *Structure* **2000**, *8* (10), 1025-1035. DOI: 10.1016/S0969-2126(00)00511-6 .

(2) Pengthaisong, S.; Piniello, B.; Davies, G. J.; Rovira, C.; Ketudat Cairns, J. R. Reaction mechanism of glycoside hydrolase family 116 utilizes perpendicular protonation. *ACS catalysis* **2023**, *13* (9), 5850-5863. DOI: 10.1021/acscatal.3c00620.
